# Linear free energy relationship between reduction potential and photoreduction rate: studies on *Drosophila* cryptochrome

**DOI:** 10.1111/febs.70129

**Published:** 2025-05-15

**Authors:** Moustafa Okasha, Jing Chen, Audrey Ayekoi, Eike Jacob, Valentin Radtke, Anton Schmidt, Adelbert Bacher, Stefan Weber, Erik Schleicher

**Affiliations:** ^1^ Institute of Physical Chemistry University of Freiburg Germany; ^2^ Institute of Inorganic Chemistry University of Freiburg Germany; ^3^ TUM School of Natural Sciences Technical University of Munich Germany

**Keywords:** cofactor exchange, cryptochrome, flavoprotein, photo‐redox reaction, radical pairs, spectroscopic characterization

## Abstract

Cryptochromes are flavin adenine dinucleotide (FAD)‐containing blue‐light photoreceptors involved in the regulation of the circadian clock and may play a role in magnetic field sensing. The photochemistry of cryptochromes is based on the isoalloxazine moiety, which can be photoreduced and subsequently reoxidized by an electron acceptor such as oxygen, corresponding to a photo‐switch between the dark and signaling state. We replaced the FAD cofactor of *Drosophila* cryptochrome with a series of FAD cofactors modified at the 7α or 8α positions, in order to modulate the chemical properties of the electron acceptor. These modifications were shown to alter the kinetics of the light‐dependent reactions. Notably, 7‐halogenated FADs form the signaling state more than six times faster compared to the natural FAD cofactor. The more positive reduction potentials as well as the increased intersystem crossing rates due to heavy halogen atoms were identified as reasons for the altered photochemistry. Both parameters show a linear dependence on the reaction kinetics, according to the Hammett relationship. With this knowledge, the photochemistry of cryptochromes may be modified in a defined way without changing its amino acid sequence.

## Graphical abstract

A series of modified FAD cofactors have been used to modulate the photochemistry of FAD‐containing cryptochrome. When 7‐halogenated FADs were used, a significantly faster signaling‐state formation was observed, due to the more positive reduction potentials and higher triplet yields compared to the natural FAD cofactors; thus, this study shows that the photochemistry in cryptochrome can be altered effectively in a wild‐type protein.

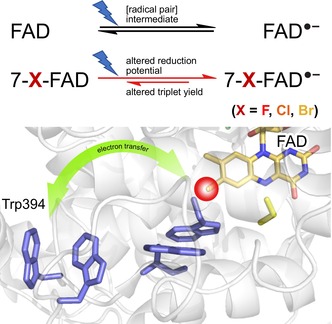

**Abbreviations**
^1^H‐NMR: proton nuclear magnetic resonance
^1^RP: singlet radical pair
^3^RP: triplet radical pair
Ag/AgCl: silver/silver chloride
*a*_iso_
: isotropic hyperfine coupling
Cry: cryptochrome
CTT: C‐Terminal tail
DmCry: *Drosophila melanogaster* cryptochrome
*E. coli*
: *Escherichia coli*
ET: electron transfer
FAD: flavin adenine dinucleotide
FMN: flavin mononucleotide
HAE: heavy atom effect
HEPES: 4‐(2‐hydroxyethyl)‐1‐piperazineethanesulfonic acid
ISC: intersystem crossing
*k*_dep_
: deprotonation rate constant
*k*_rec_
: recombination rate constant
*k*_red_
: reduction rate constant
LF: lumiflavin
RF: riboflavin
RP: radical pair
TA: transient absorption
Tr‐EPR: transient electron paramagnetic resonance
X: halogen atoms

## Introduction

Cryptochrome photoreceptors evolved from light‐dependent DNA repair enzymes designated as photolyases [1, 2] and have been characterized from all domains of life [3]. They are known to function as components of the circadiurnal clock [4, 5] and may also be involved in magnetic field sensing [6, 7, 8, 9, 10].

**Fig. 1:**
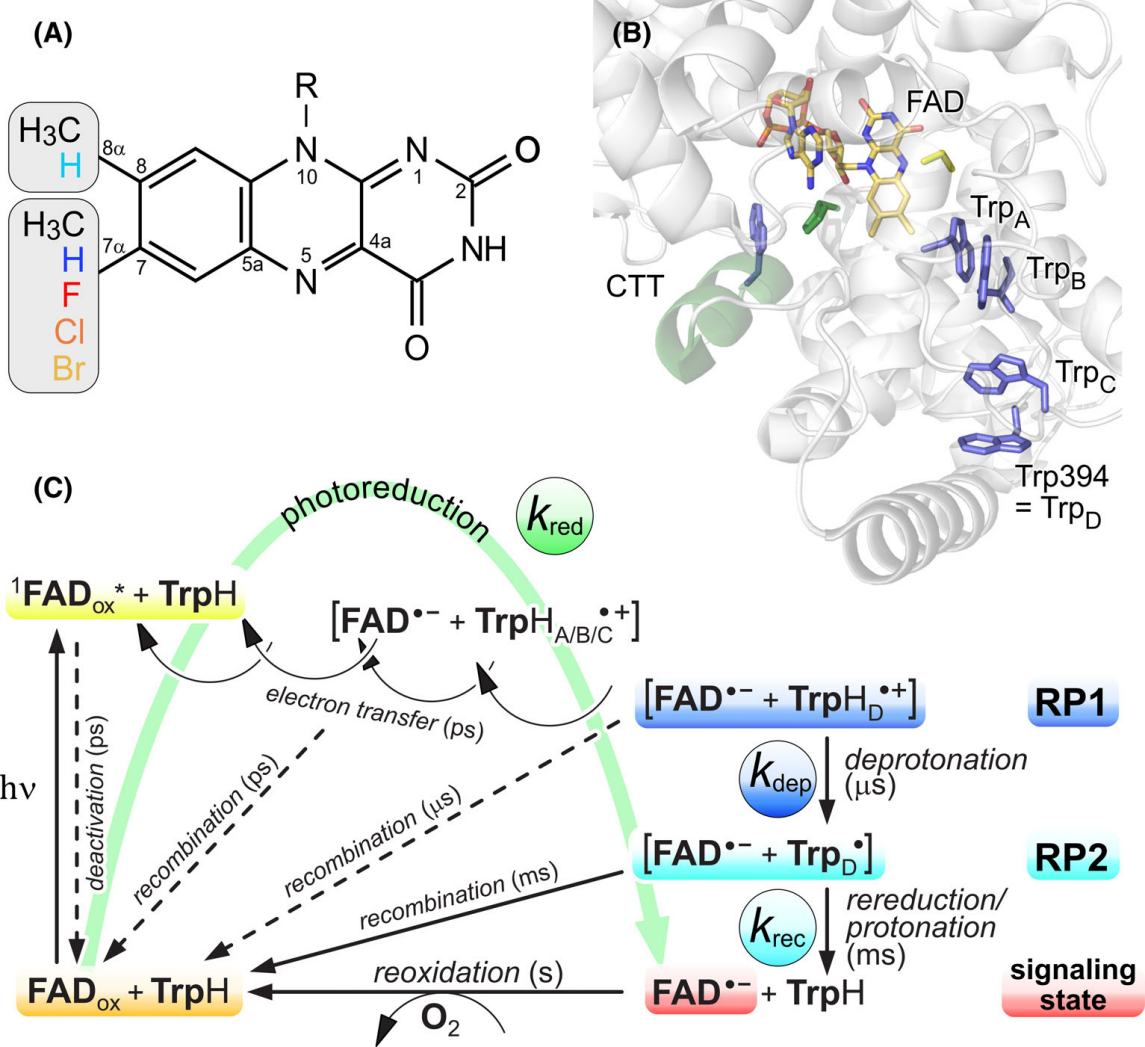
Overview of the photoreactions in DmCry. (A) Structure, IUPAC numbering scheme, and modifications of the flavins used in this study. (B) Important regions of DmCry: the CTT (green), the FAD cofactor (orange), and the Trp‐tetrad (blue) (protein structure was generated using Pymol). (C) Photochemical reactions in DmCry and their approximate time scales. The terminal surface‐exposed [FAD^·−^···TrpH_D_^·+^] radical pair (RP1) is stabilized by deprotonation forming RP2, and after rereduction of the Trp_D_^·^ radical, the metastable FAD^·−^ signaling state is formed [generated using affinity designer 1.10, Serif (Europe) Ltd., Nottingham, UK].

The active site of the *Drosophila melanogaster* cryptochrome (DmCry) [11, 12] including important amino acids (Trp‐tetrad) is shown in Fig. 1. Its helical C‐terminal tail (CTT) plays an important role for photoreception as it is attached to the protein in the dark, thus preventing interaction and, in turn, enabling interaction with effector proteins after light excitation [13].

The photoreaction of cryptochrome involves the transfer of an electron from a nearby tryptophan side chain (Trp_A_) to the FAD cofactor, conducing to the generation of a spin‐coupled [FAD^·−^···TrpH_A_^·+^] radical pair (RP) (Fig. 1C). Subsequently, stepwise sequential electron transfer (ET) takes place along a chain of conserved tryptophans [14, 15] until the terminal surface‐exposed tryptophanyl cation radical is formed (RP1). The charged TrpH^·+^ radical then deprotonates within microseconds (denoted as rate constant *k*_dep_) [15, 16, 17]; this secondary radical pair (RP2) can recombine back to the ground state, making the RP reaction cyclic (denoted as rate constant *k*_rec_). Alternatively, the tryptophan radical is reduced by an external electron donor, resulting in a net reduction of the FAD cofactor, the so‐called photoreduction reaction (denoted as steady‐state rate constant *k*_red_). ET in DmCry takes place along a tetrad of tryptophans (denoted as TrpH_A/B/C/D_, Fig. 1B); the resulting semireduced FAD is mostly in its unprotonated FAD^·–^ state [18, 19, 20, 21]. The FAD radical state has been shown to act as a signaling state in plant and animal Cry photoreceptors [22, 23]. In DmCry, the time scale of conformational changes upon signaling‐state formation proceeds 5 ms after blue‐light excitation [24].

In the signaling state, conformational changes of the CTT enable interaction between Cry and the *timeless* protein, which suppresses the feedback loop of the internal clock [25, 26, 27, 28]. Moreover, there is evidence that DmCry mediates a range of magnetic‐field‐dependent phenotypes in fruit flies [29, 30, 31] although the exact impact is still under discussion [32, 33, 34, 35]. It was also shown that purified protein shows altered reaction kinetics under the influence of weak magnetic fields *in vitro* [36]. The underlying mechanism for this magnetic field effect is assumed to be the so‐called radical pair mechanism [7, 8].

The common approach to studying light reactions of Crys was to examine the protein from different organisms and/or to replace the photochemically relevant amino acids in order to quantify the altered reactivity and/or selectivity (e.g., [13, 19, 37]). However, this approach has the fundamental constraint that the amino acids Trp and Tyr used as electron donors in biological ET can only be replaced by the similarly sized, but redox‐inactive amino acid Phe, and that the electron acceptor is always the FAD cofactor. To circumvent these limitations, the photophysics may be modulated on the FAD side by the incorporation of modified flavins. In principle, the photophysics can also be modulated by the incorporation of unnatural amino acids that alter the electron donor site [38]. There are a number of potential candidate molecules, but as both size and reduction potential need to be similar to Trp, the concept is not as straightforward and has therefore not yet been realized in Crys. Various methods for replacing protein cofactors have been established, typically by partial denaturation and subsequent refolding of the protein [39, 40]. However, the available methods have not been successful with cryptochromes.

We report a method for the preparation of DmCry loaded with modified FAD cofactors by *in vivo* expression [41, 42]. Spectroscopic analysis established a linear free energy relationship between the reduction potential of the flavins carrying different substituents at positions 7α or 8α (Fig. 1A and Fig. S1) and the photoreaction rates of the cognate cryptochrome apoproteins.

## Results

### *In vivo* incorporation of modified flavins into DmCry

Chemically modified FADs were incorporated into DmCry using a riboflavin‐auxotrophic *E. coli* strain [41, 42]. To analyze the incorporation yields of the modified flavins and to determine how their optical properties change upon incorporation, the UV–Vis spectra of protein‐bound and free modified FADs/RFs were compared with those of unmodified FAD/RF to identify any characteristic shifts in the absorption bands. In general, the optical spectrum of RF is characterized by two absorption maxima around 445 and 375 nm. The former maximum is mainly assigned to the S_0_ → S_1_ electronic transition while the S_0_ → S_2_ and higher electronic transitions contributes to the band at 375 nm [43]. A comparison of the absorption spectra of the two demethylated RF derivatives, 7‐demethyl‐RF and 8‐demethyl‐RF, with those of free RF showed a 14‐nm blue shift of the S_0_ → S_1_ transition in the former, and a 21‐nm blue shift of the S_0_ → S_2_ transition in the latter. Similar to 8‐demethyl‐RF, 7‐halogen‐RFs (7‐X‐RF) derivatives (X = F, Cl, Br) did not show any significant shift of the S_0_ → S_1_ transition but have a ≈17 nm blue shift of the S_0_ → S_2_ transition (Fig. S2 and Table S1).

**Fig. 2:**
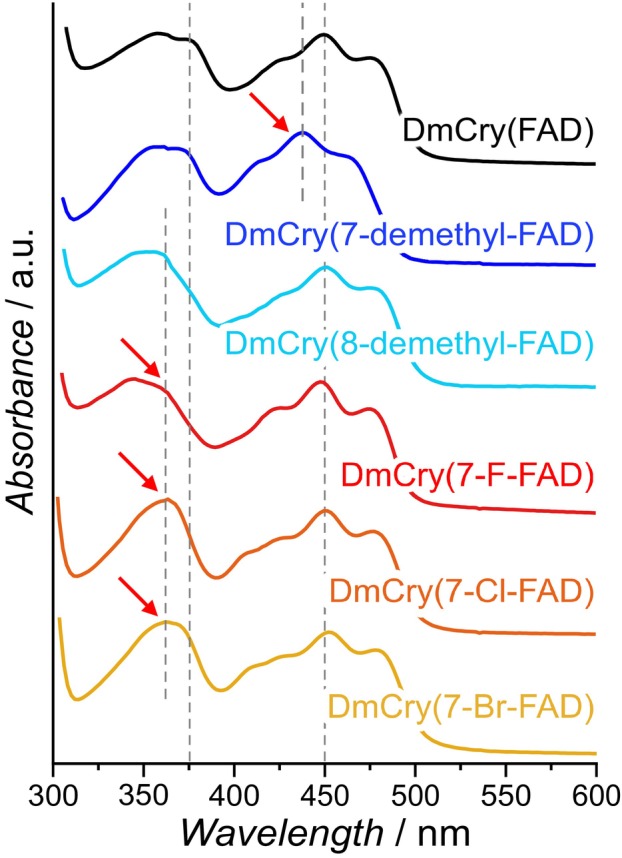
UV–Vis spectra of DmCry with unmodified (black) and modified FAD cofactors, recorded under aerobic conditions in the FAD_ox_ state. Sample concentrations were *c* = 50 μm. The two long‐wavelength maxima of FAD are shown as vertical dashed lines, the thick dashed lines represent exemplary blue shifts of the maxima for modified FADs. Spectral shifts are highlighted by red arrows.

After incorporation of the (unnatural) flavin derivatives into DmCry, all samples show the characteristic vibration fine structure of protein‐bound flavins, namely shoulders at 430 and 470 nm (Fig. 2). In addition, all of them exhibit absorption maxima shifts very similar to those of the modified RFs in solution described above: DmCry(7‐demethyl‐FAD) shows a blue shift of the S_0_ → S_1_ band, while the other modified flavins show only a blue shift of the S_0_ → S_2_ band. In addition, the S_0_ → S_1_ band of DmCry(7‐Cl‐FAD) and DmCry(7‐Br‐FAD) is red‐shifted by a few nm. Thus, the incorporated modified FADs investigated in this study can be distinguished from unmodified FAD by their absorption spectra.

The *in vivo* incorporation process critically relies on supplementing the RF auxotrophic *E. coli* strain with synthesized RF. As LB medium contains some RF (0.08% per culture flask), there is a possibility that RF from the medium may be incorporated into the produced DmCry, which would reduce the incorporation yield of the modified FADs. Therefore, additional analyses were performed to confirm that only modified flavins were incorporated: First, modified and unmodified DmCry protein samples were denatured, and the extracted FAD cofactors were compared to the respective free modified RFs by UV–Vis spectroscopy (Fig. S2), showing that the respective absorption peaks and their shifts with respect to unmodified RF match very well for all samples. Secondly, HPLC analysis of one of the extracted modified FAD cofactors (from DmCry(7‐demethyl‐FAD)) was performed (Fig. S3). Two fractions were collected with a very short retention time difference. Because of this small difference and the almost identical absorption maxima (Fig. S3B), the second fraction is believed to be 7‐demethyl‐FMN degraded from 7‐demethyl‐FAD during the extraction protocol. This suggestion was confirmed by ^1^H‐NMR spectroscopy, which identified the two fractions as 7‐demethyl‐FAD and 7‐demethyl‐FMN (Fig. S3C) based on the missing signal of the 7α‐methyl group at around 2 ppm and the appearance of two doublets of proton ortho‐coupling of C6 and C7 at 7.5 and 7.9 ppm, respectively, and one singlet peak of the C9‐proton at 7.8 ppm. Additionally, two singlet resonances were assigned to the two aromatic protons (attached to C2 and C8) of the adenine group. The ^1^H‐NMR spectrum of the second fraction showed similar signals of the isoalloxazine moiety; however, the two singlet resonances of the adenine protons are absent, which is in line with a reference sample of 7‐demethyl‐FMN. Unmodified flavin could not be detected. It can therefore be assumed that all produced samples contain a sufficiently high fraction of modified flavins suitable for further spectroscopic investigations.

### Accelerated photoactivation of modified DmCrys

**Fig. 3:**
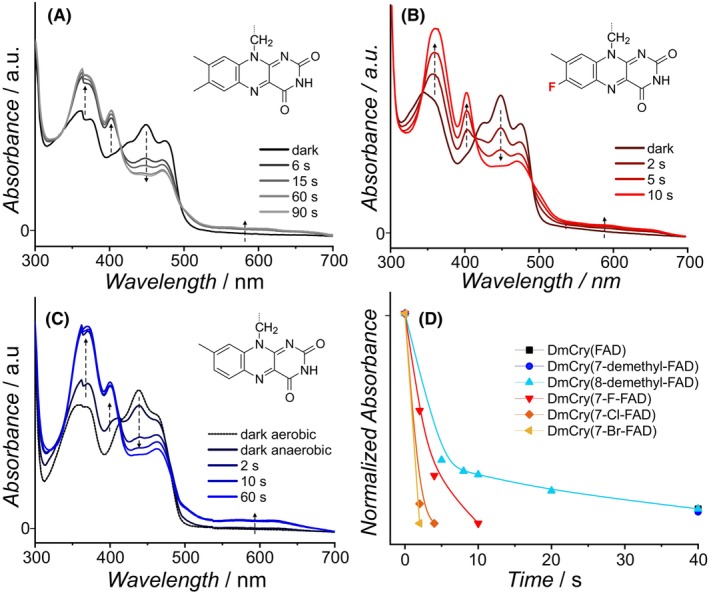
UV–Vis spectra of the photoreduction of DmCry samples. (A) Photoreduction of unmodified DmCry. Exemplary modified samples of DmCry(7‐F‐FAD) (B) and DmCry(7‐demethyl‐FAD) (C). Illumination times are indicated in the respective panel, additional photoreductions are depicted in Fig. S4. (D) Time‐dependent absorption decay at 450 nm (corrected to the absorbance maxima) of indicated samples. Sample concentrations were  *c* = 50 μm. Vertical dashed arrows indicate the increase or decrease of the respective absorption bands.

The photoreduction of DmCry is essentially a light‐induced one‐electron reduction of FAD_ox_ to the anionic semiquinone FAD^·−^ state (Fig. 1C) [17, 18]: the intermediately formed RP2 can either recombine, or the surface‐exposed Trp394 (Trp_D_) radical is reduced by an external electron donor during the lifetime of RP2; in the latter case, the metastable FAD^·−^ radical remains unchanged. UV–Vis spectroscopy was used to analyze the photoreduction kinetics, whereby a decrease of the FAD_ox_ absorption band at 450 nm is expected along with an increase of FAD^·−^ signals with peaks at 372, 402, and 470 nm. All DmCry samples with modified FAD cofactor were photoactive and performed the photoreduction reaction (Fig. 3 and Figs S4 and S5); only small intensity changes and shifts of the respective FAD^·−^ absorption spectra maxima can be detected (Fig. S5). In particular, this applies to the DmCry(7‐Cl‐FAD^·−^) and DmCry(7‐Br‐FAD^·−^) spectra, in which the two long‐wavelength maxima are red‐shifted by ~ 5 nm.

Depending on the sample, a small percentage of protonated FADH^·^ state, which has absorbance in the (550–650)‐nm region, is additionally detected (Fig. 3 and Fig. S4D). In DmCry, the amount of FADH^·^ formation was found to be strongly pH‐ and buffer‐dependent, ranging from ≈10% to 25% [21]. Under the experimental conditions used in this study, 13.5% of the FADH^•^ state is formed. While the fraction of the protonated radical in DmCry(7‐demethyl‐FAD) is almost unchanged (15.7%), the incorporation of 7‐halogenated FADs leads to significant changes: the fraction of 7‐X‐FADH^•^ increased in the order DmCry(7‐Br‐FAD) (0%) via DmCry(7‐Cl‐FAD) (5.2%) to DmCry(7‐F‐FAD) (11.3%). These changes may originate from different p*K*_a_ values of the individual flavin radicals, as we assume that the FAD binding position and its protein environment remain unchanged.

The photoreduction rate constants (*k*_red_) with EDTA as an external electron donor were analyzed using monoexponential decay functions for fitting the experimental values of all 7‐modified FAD samples (Fig. 3D and Table 1). We were interested in the extent to which changes in the 7α position can influence the photochemistry and whether each modification causes similar changes. DmCry(7‐demethyl‐FAD) resulted in a 1.7‐fold higher photoreduction rate compared to unmodified DmCry. Even more pronounced differences of *k*_red_ were observed in the three halogenated FAD samples: DmCry(7‐F‐FAD) shows an increase by a factor of 2.7, for DmCry(7‐Cl‐FAD) and especially for DmCry(7‐Br‐FAD) the rates increase by a factor of 3.1 and > 6.1, respectively. The uncertainty of the latter value is rather high as the 7‐Br‐FAD^·−^ formation is already completed within the shortest illumination interval of 2 s, and this rate may therefore be significantly higher. In sum, *k*_red_ of DmCry(7‐X‐FAD) samples increases in the order F < Cl < Br.

**Table 1:** Photochemical rate constants of all investigated DmCry samples. Photoreduction (*k*_red_) was determined by optical spectroscopy (Fig. 3 and Fig. S4) and monoexponential decay functions at 450 nm. The TA rate constants (*k*_dep_ and *k*_rec_), extracted from Figs S8–S11, were determined by monoexponential decays at 580 and 520 nm, respectively. Error margins represent standard deviations.

	Photoreduction kinetics	Radical pair kinetics extracted from TA spectroscopic analyses		
	*k*_red_ /s^−1^	*k*_dep_ /μs^−1^	*k*_rec_ (Trp_D_^·^) /ms^−1^	*k*_rec_ (FAD^·−^) /ms^−1^
DmCry(FAD)	0.28 ± 0.06	0.27 ± 0.005	0.25 ± 0.005	0.25 ± 0.01
DmCry(7‐demethyl‐FAD)	0.48 ± 0.08	0.24 ± 0.06	0.24 ± 0.06	–
DmCry(8‐demethyl‐FAD)	0.33 ± 0.09	0.26 ± 0.003	0.20 ± 0.03	0.20 ± 0.03
DmCry(7‐F‐FAD)	0.77 ± 0.5	0.23 ± 0.01	0.25 ± 0.02	No decay
DmCry(7‐Cl‐FAD)	0.89 ± 0.3	0.25 ± 0.01	0.23 ± 0.02	No decay
DmCry(7‐Br‐FAD)	> 1.70 ± 0.6	0.26 ± 0.03	0.28 ± 0.03	No decay

The photoreactivity of DmCry(7‐Br‐FAD) is so high that even under low‐light conditions using blue‐light filters, which are used during purification and sample preparation, significant FAD reduction takes place. To investigate this finding in more detail, DmCry(7‐Br‐FAD) was incubated under anaerobic conditions for different times under low‐light conditions, and the formation of (7‐Br‐FAD)^·−^ could already be detected after 5 min (Fig. S4C). Even under aerobic conditions, DmCry(7‐Br‐FAD)^·−^ is stable for several hours. Reoxidation with air of the three DmCry(7‐X‐FAD) samples is only fully completed after overnight incubation (Fig. S6).

### Different chemical properties of the modified DmCry samples

**Fig. 4:**
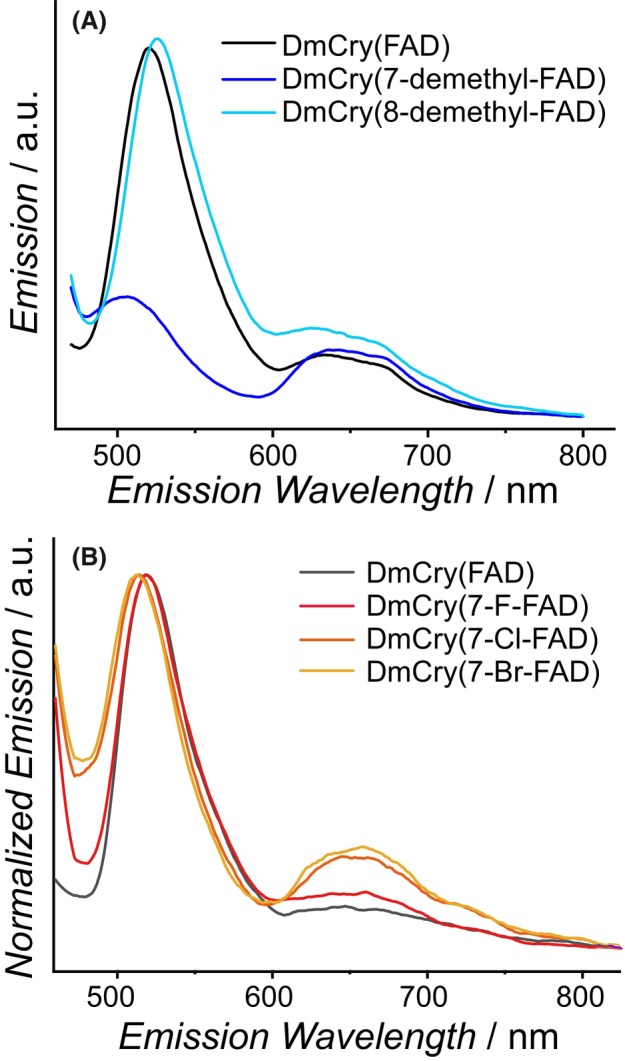
Emission spectra of DmCry samples using an excitation wavelength of 440 nm. (A) Emission spectra of DmCry(7‐demethyl‐FAD) (blue) and DmCry(8‐demethyl‐FAD) (light blue) compared to DmCry(FAD) (black). (B) Normalized emission spectra of DmCry(7‐F‐FAD) (red), DmCry(7‐Cl‐FAD) (orange) and DmCry(7‐Br‐FAD) (yellow) compared to DmCry(FAD) (black). Sample concentrations were *c* = 50 μm.

Emission spectra of unmodified and modified flavins, both in solution (Fig. S7) and incorporated into DmCry (Fig. 4), were recorded after excitation at 440 nm. The emission spectrum of free RF exhibits broad fluorescence between 500 and 580 nm with a maximum at 522 nm (Table S1). In comparison, the fluorescence intensity of 8‐demethyl‐RF is slightly increased, and the emission maximum of 7‐demethyl‐RF is 11 nm blue‐shifted, accompanied by a lower emission intensity. The latter effect could indicate that the lifetime of the excited singlet state is reduced or that the absorption coefficient or the fluorescence quantum yield is lower.

In addition to the fluorescence signal, DmCry‐bound FAD shows a weak emission between 620 and 680 nm, which can be assigned to phosphorescence from the triplet excited state (^3^FAD*). The two samples DmCry(7‐demethyl‐FAD) and DmCry(8‐demethyl‐FAD) show a dissimilar behavior: While the incorporation of 8‐demethyl‐FAD shows almost no changes compared to DmCry(FAD), the fluorescence peak of the DmCry(7‐demethyl‐FAD) sample is 10 nm blue‐shifted and the fluorescence intensity is reduced by about 70% (Fig. 4A). Different changes in the emission properties are observed in the three DmCry(7‐X‐FAD) samples: While a decrease of the fluorescence intensities is observed in the order 7‐F‐FAD > 7‐Cl‐FAD > 7‐Br‐FAD (Fig. S7B), the phosphorescence signal centered at 650 nm increases in the order 7‐F‐FAD < 7‐Cl‐FAD < 7‐Br‐FAD (Fig. 4B). This finding indicates an enhanced intersystem crossing (ISC) probability and thus an increased formation of the metastable ^3^[7‐X‐FAD]* state by the introduction of halogen atoms at the 7α‐position.

Another reason for the altered photoreduction kinetics may result from different redox potentials of the modified flavin derivatives. Cyclic voltammetric measurements were hence performed for RF derivatives and compared with published values of the corresponding lumiflavin (LF) derivatives (vs. Ag/AgCl, Table 2) [44]. RF shows an increase in the reduction potential by +45 mV in comparison to LF (−411 vs. –456 mV). This increase in reduction potential can be observed in a direct comparison of the respective modified RF derivatives and modified LF derivatives. If the reduction potentials of the individual modifications are compared with those of RF (or LF), a differentiated picture emerges: all modified flavins have a more positive reduction potential, with the smallest change of +19 mV being observed in 7‐demethly‐RF and the largest of over +50 mV in 7‐Cl‐RF and 7‐Br‐RF. Given that the FAD modifications do not lead to a relevant change in cofactor binding, a similar reduction behavior of the bound cofactors can be assumed.

**Table 2:** Reduction potentials (*E*°, vs. NHE) of indicated RF derivatives compared to RF in buffered aqueous solution in comparison to published LF derivatives, extracted from [31].

Flavins	*E*° (RF derivatives)	Δ*E*° (vs. RF)	*E*° (LF derivatives)	Δ*E*° (vs. LF)
RF/LF	−214 mV	0	−259 mV	0
7‐demethyl‐	−195 mV	+19 mV	−242 mV	+17 mV
8‐demethyl‐	−170 mV	+44 mV	−222 mV	+37 mV
7‐F‐	−175 mV	+39 mV	−201 mV	+58 mV
7‐Cl‐	−160 mV	+54 mV	−205 mV	+54 mV
7‐Br‐	−156 mV	+58 mV	n.d.	n.d.

**Fig. 5:**
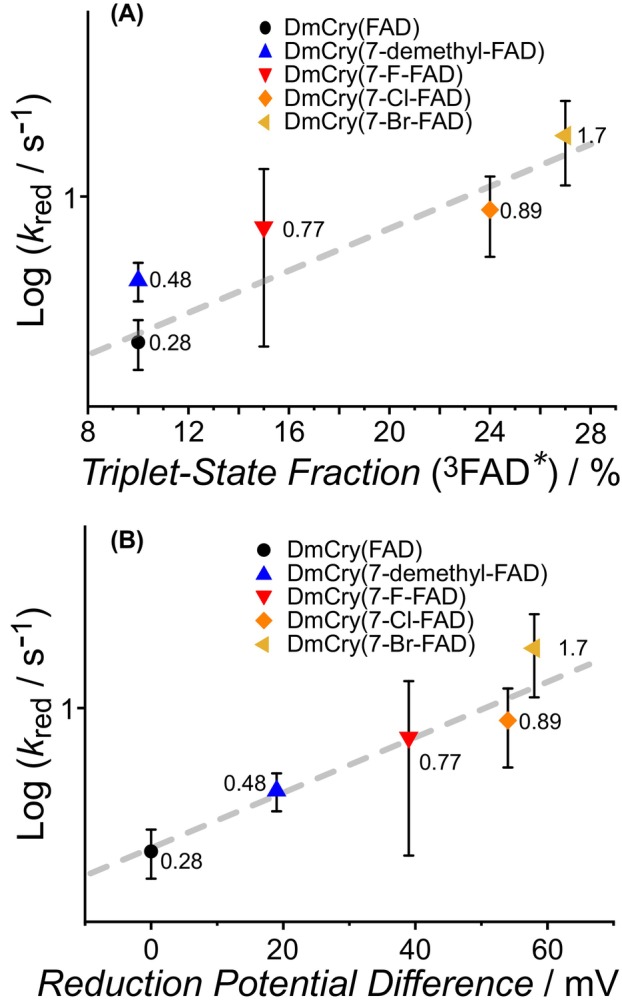
Photoreduction dependence on triplet state fractions and reduction potential difference. Dependence of photoreduction rate *k*_red_ versus (A) the triplet state fractions extracted from Fig. 4, and (B) the reduction potential difference of the modified flavins measured in solution. Gray dashed lines are to guide the eye. Error bars represent the standard deviation (SD).

For more detailed analyses of the influence of the respective 7α cofactor modification, the photoreduction rate constants were plotted against the generated triplet fraction on the one hand and against the reduction potential in solution on the other (Fig. 5A,B). A similar relationship can be observed in both plots: The CH_3_‐to‐H exchange leads to only a small increase in log(*k*_red_), while log(*k*_red_) increases in the order F < Cl < Br for the three halogenated samples, regardless of whether it is plotted against the triplet fraction or the reduction potential. Both of these diagrams can generally be interpreted in such a way that there is a direct relationship between the rate constant and the substituents.

### Radical pair dynamics of modified DmCrys

To gain further insight into the molecular processes induced by blue‐light illumination, transient absorption (TA) spectroscopy with nanosecond time resolution was used. DmCry proteins were photoexcited by a laser pulse at 460 nm, and transient absorbance changes were monitored over a wavelength range from 370 to 700 nm for either micro‐ or milliseconds (Figs S8–S11). Following previous analyses of the DmCry photochemistry [17, 19, 21], RP1 deprotonates on the μs‐time scale to form RP2, which recombines on the ms‐time scale (Fig. 1B). Qualitatively, DmCry difference spectra that were recorded starting from the FAD_ox_ state can be divided into different parts, namely a negative band at around 450 nm that is assigned to FAD_ox_ ground‐state bleaching, and positive difference bands in the (375–415) nm and (500–550) nm regions that arise from the [FAD^·−^···Trp_D_^·^] RP and TrpH_D_^·^ signals in the (560–650) nm region. By using exponential decay functions at specific wavelengths, accurate rate constants for formation (*k*_dep_) and decay of RP2 (*k*_rec_) were obtained (Table 1). DmCry(FAD) exhibits rate constants *k*_dep_ = 0.27 μs^−1^ and *k*_rec_ = 0.25 ms^−1^ under the chosen experimental conditions, which are very similar to published values [17, 19, 21].

In comparison, the time profiles of DmCry(7‐demethyl‐FAD) show no significant changes in the rate constants, neither in the μs‐ nor in the ms‐time scale (Table 1). The TA measurements of DmCry samples with 7‐X‐FAD cofactors exhibited the special feature that the first scan showed clear difference signals, while no signals could be detected in a second scan (Fig. S12). This finding can be rationalized by the efficient photoreduction and thus a significant decrease in the FAD_ox_ concentration after each laser flash. Additional fluorescence spectra of the DmCry(7‐X‐FAD) samples were measured to document the formation of 7‐X‐FAD radicals; a representative measurement of DmCry(7‐Cl‐FAD) is shown as Fig. S13. After 10 s of blue‐light illumination, the fluorescence intensity is already reduced by about 50% compared to DmCry(FAD), which can be attributed to the simultaneous accumulation of 7‐X‐FAD^·−^ and reduction of the FAD_ox_ state. As the FAD reoxidation takes several hours to complete (see above), freshly prepared samples were used for each scan.

**Fig. 6:**
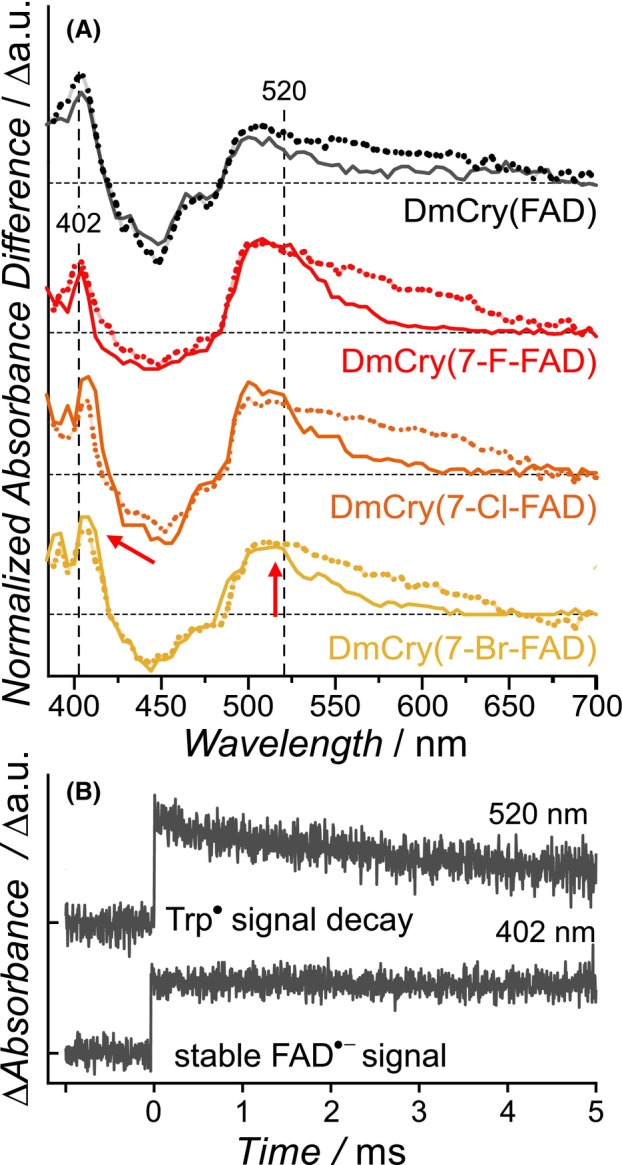
Transient absorption spectra of modified DmCry samples. (A) Difference absorption spectra of DmCry with indicated cofactors, recorded in the μs‐ (dashed lines) and ms‐time scale (solid lines). Spectral shifts are marked by red arrows, vertical dashed lines indicate the wavelengths at which the time decays are shown in (B). (B) Exemplary decay of RP2 signals of DmCry(7‐Cl‐FAD) at 402 nm (mostly absorption of FAD^·−^) and 520 nm (mostly absorption of Trp_D_^·^). Full 2D datasets are depicted as Figs S8–S11.

In general, the obtained difference absorption spectra of the DmCry(7‐X‐FAD) samples show very similar curves (Fig. 6A). Slight changes in minima/maxima are due to changes in the absorption properties of the respective modified FAD_ox_ and FAD^·−^ states (Fig. 2 and Fig. S5). Analysis of the time traces reveals that the decay of the TrpH^·+^ signals (*k*_dep_) has very similar rates compared to DmCry(FAD) (Table 1). Analysis of the decay of RP2 (*k*_rec_) turned out to be difficult for DmCry(7‐X‐FAD) samples as a significant amount of difference signal persisted within the 6 ms‐time scale of the experiments. Performing the analysis at two wavelengths, at which mostly Trp^·^ (520 nm) and FAD^·−^ (402 nm) absorb, revealed that Trp^·^ is reduced with a similar time constant as DmCry(FAD), whereas FAD^·−^ does not decay in all three (7‐X‐FAD)^·−^ samples (Fig. 6B depicts an exemplary time trace of DmCry(7‐Cl‐FAD^·−^)). The quantification of the RP2 decay and its unchanged fraction clearly shows that the RP recombination of the DmCry(7‐X‐FAD) samples is asynchronous.

### Transient EPR spectroscopy of DmCry with modified FADs

Transient electron paramagnetic resonance (trEPR) spectroscopy recorded in direct‐detection mode allows the observation of short‐lived RPs and triplet states on a nanosecond time scale [45]. Consequently, positive and negative signal amplitudes in trEPR correspond to enhanced absorptive (A) and emissive (E) electron spin polarization of the EPR transitions, respectively. In general, from a trEPR spectrum, information on the excited state, such as the Zeeman interaction, hyperfine couplings, spin–spin interactions in the case of more than one paramagnetic center, and the zero‐field splitting (in case of a high‐spin system with *S* > ½), is obtained.

Various proteins from the Cry/Photolyase family, including DmCry, have been investigated previously using the trEPR method [19, 46, 47, 48, 49]. DmCry exhibits a complex spectral pattern that has been assigned to a [FAD···Trp_D_] RP with a distance of 2.15 nm. For spectral simulations, several hyperfine couplings, both from the donor and the acceptor, and values of −0.285 mT and 5 μT for the two electron–electron interaction parameters *D* and *J*, respectively, had to be included to reproduce the experimental data [19].

**Fig. 7:**
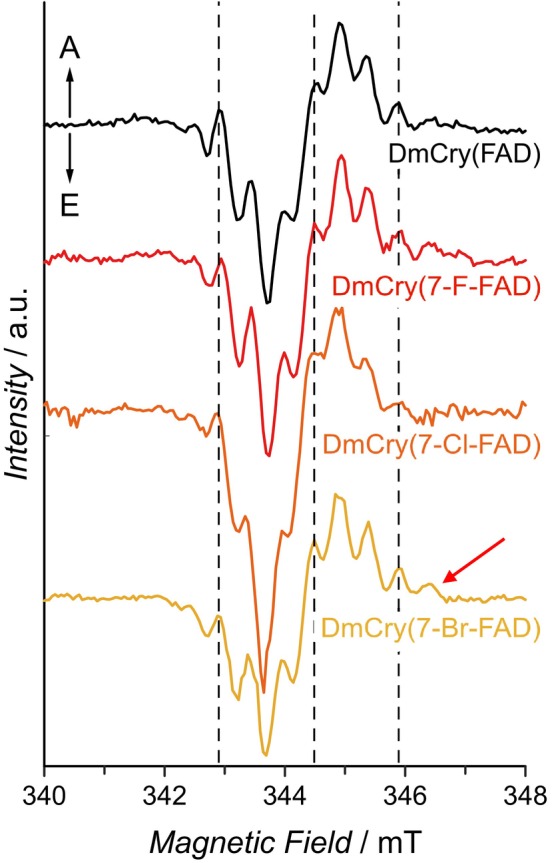
Normalized trEPR spectra of unmodified and modified DmCry samples (1D spectra were generated by integrating from about 310 to 1210 ns in the respective time domains). Both DmCry(FAD) and all DmCry(7‐X‐FAD) samples show the formation of an RP. Additional signals with respect to the DmCry(FAD) sample are indicated by a red arrow. All spectra were corrected to a microwave frequency of 9.65308 GHz.

The trEPR spectra of the three DmCry(7‐X‐FAD) samples measured at X‐band microwave frequency show the characteristic E/A pattern of a spin‐correlated RP superimposed with a complex hyperfine splitting (Fig. 7, full datasets are shown as Fig. S14). Unexpectedly, the signals do not differ significantly from those of the DmCry(FAD) sample at first glance: maxima and minima of the individual signals are at identical positions, only the signal intensities are slightly different for a few signals, which may be due to a different hyperfine coupling pattern as well as slightly different background signals. Another possibility would be that a small fraction of a ^3^RP, generated from a triplet precursor (analogous to Fig. 4), contributes to the trEPR spectrum. A ^3^RP exhibits an inverted polarization pattern with an identical hyperfine coupling pattern [47], so the resulting signal should have a lower signal intensity due to partial cancelation. Unfortunately, too many parameters such as the sample concentration or the effective laser excitation of the sample affect the trEPR signal intensity, so that the contribution of the ^3^RP cannot be quantified.

Upon closer inspection, however, the DmCry(7‐Br‐FAD) sample shows an additional signal at 346.5 mT (arrow in Fig. 7). When replacing the 7α‐methyl group with halogens, a change in the signal intensity and in the strength of the hyperfine coupling is expected. In unmodified FAD, the signals of the three methyl protons (*I* = 1/2) add up to a set of magnetically equivalent protons [50]; after fluorine (*I* = 1/2) exchange, three proton signals change to one fluorine signal, which should lead to a decrease in signal intensity. In addition, the electron spin density of the protons and fluorine should differ, since the former are attached to C7α and the latter is attached to C7. The situation becomes more complex for chlorine as two isotopes (^35^Cl and ^37^Cl with 75/25% natural abundance), both with a nuclear spin of *I* = 3/2, should complicate the hyperfine pattern significantly. The same holds true for bromine exchange as both naturally occurring isotopes, ^79^Br and ^81^Br with similar natural abundances, are *I* = 3/2 nuclei. Most importantly, the strength of the hyperfine coupling depends directly on the electron spin density at the corresponding nucleus. The spin density at position 7 (and at position 7α) is quite small, leading to an experimentally determined small hyperfine coupling of *a*_iso_≈2 MHz for a 7α‐methyl group [51]. It is therefore likely that the effect of the cofactor exchange on the hyperfine coupling pattern is quite small as long as the spin density does not change by halogen incorporation.

Spectral simulations of these spectra are challenging due to the substantial number of hyperfine couplings. However, the signal patterns exhibited by the samples DmCry(7‐Cl‐FAD) and DmCry(7‐Br‐FAD) are distinguishable, despite the presence of an additional *I* = 3/2 nucleus in both. The most probable interpretation is that the presence of different halogen atoms results in altered spin density distributions and consequently small variations in the hyperfine patterns, which in turn give rise to an additional signal.

To learn more about the spin density distribution of the DmCry(7‐X‐FAD) samples and perhaps gain useful hyperfine couplings as starting values for spectral simulations, quantum chemical calculations at the DFT theory level were performed using 7‐X‐FMN in a water surrounding. The results show that the hyperfine couplings of the respective isoalloxazine backbones are very similar (Table S2), only the hyperfine coupling of 7‐F‐FMN is larger than that of 7‐Cl‐FMN and 7‐Br‐FMN. This unexpected result can only be explained by hyperconjugation, as the spin densities of the individual halogen atoms are similar to that of the 7α‐methyl group, and the spin densities change only slightly (Mulliken spin populations: −1.03 × 10^−3^ (7‐CH_3_‐FMN), −0.65 × 10^−3^ (7‐F‐FMN), −1.38 × 10^−3^ (7‐Cl‐FMN) and −1.23 × 10^−3^ (7‐Br‐FMN)). Because no change in the coupling pattern can be detected for DmCry(7‐F‐FAD) compared to the other samples, the influence of the 7α position on the coupling pattern is obviously not large enough to be easily detectable in trEPR spectra.

## Discussion

### Incorporation of modified cofactor

Methods for replacing cofactors in flavoproteins have been used for several decades [40, 52]. Most of them are based on partial protein denaturation upon lowering the pH or by adding chaotropic salts. Under these conditions, the noncovalently bound (native) cofactor can be released from the binding pocket and extracted. The apoprotein is then reconstituted by the addition of a modified cofactor under appropriate conditions. Although such techniques allowed the successful incorporation of modified cofactors into small light‐active flavoprotein domains [53] and different flavoenzymes [54, 55, 56, 57], protocols on cofactor exchange in photolyases or Crys have not been published so far.

To incorporate modified flavins into DmCry, a riboflavin‐auxotrophic *E. coli* strain was employed. This strategy has already proven effective in substituting cofactors in small flavoprotein photoreceptors [41, 42]. Five different modified flavins were incorporated into DmCry, and analysis of these samples confirmed their successful incorporation (Fig. 2 and Figs S2 and S3).

Although the protein yields of the individual samples differed and were lower than those obtained from unmodified DmCry, sufficient amounts of sample material for spectroscopic studies were obtained. With the exception of the DmCry(8‐demethyl‐FAD) sample, which showed reduced stability and hence it was difficult to obtain good signals in TA spectroscopy (Figs S9B and S11), the protein stability was comparable to that of DmCry(FAD). Thus, even for larger proteins with a central cofactor‐binding pocket, *in vivo* incorporation can be a very efficient alternative compared to conventional refolding protocols. Some limitations of this method exist particularly for highly modified flavins: Certain modified flavins may be toxic to the host organism. Information on the promiscuity of the flavin importer system used, the riboflavin transporter RibM from *C. glutamicum*, is scarce [42], so it is likely that not all modified flavins are transported into the cell with high yield. In addition, steric hindrances may prevent successful uptake, particularly for flavins with highly different geometry or spatial dimensions. In the case of DmCry, incorporation of flavin derivatives with a bulky group attached to C8 may be challenging as the amino acid His_378_ is close and is thought to play a critical role in C‐terminal displacement and signaling, although the exact molecular mechanism is still under investigation [21, 58].

### Spectroscopic characterization

Spectroscopic information on the modified DmCry samples was obtained from steady‐state photoreduction experiments as well as the application of two time‐resolved techniques, trEPR and TA spectroscopy. DmCry samples with cofactors modified at the C7α‐position show significantly different signaling‐state formation kinetics, both compared to an unmodified sample and relative to each other (Table 1). The kinetics increase in the order CH_3_ < H < F < Cl < Br up to a factor of > 6. DmCry(7‐Br‐FAD) is efficiently photoreduced even under minimal light conditions without the addition of an electron donor; the resulting (7‐Br‐FAD)^·−^ is stable for several hours under aerobic conditions. As the protein environment and accordingly the accessibility of oxygen remain unchanged, the enhanced stability of the anion radical points to an altered redox potential.

To rationalize the enhanced photoreduction, emission spectroscopy and the reduction potentials of the modified flavins were determined. The emission spectra of the modified flavins show different effects dependent on the modification: While DmCry(8‐demethyl‐FAD) shows essentially no difference to unmodified FAD, the fluorescence of DmCry(7‐demethyl‐FAD) is virtually no longer recognizable (Fig. 4). This may be due to a reduced lifetime of the excited state *(7‐demethyl‐FAD) as a result of more effective ET, or this cofactor modification may also favor a different non‐radiative deactivation channel. The faster photoreduction (see above), which is accompanied by a higher ET probability, indicates that a reduced excited state lifetime is probably not the only reason for the low fluorescence yield. The emission spectra of DmCry(7‐X‐FAD) show a significant increase in the phosphorescence emission of the triplet state in the DmCry(7‐Br‐FAD) and DmCry(7‐Cl‐FAD) samples, and to a lower extent in DmCry(7‐F‐FAD) (Fig. 4), indicating an enhanced ISC rate of the three samples. Even though transitions between pure spin states of different multiplicity are generally forbidden due to the spin selection rule, there are numerous examples of such transitions since the spin‐orbit coupling leads to a quantum mechanical mixing of these spin states [59]. The spin‐orbit coupling results qualitatively from the interaction between the magnetic moment of the electron spin and the electrostatic field of the positively charged nucleus. As the strength of the latter magnetic field is directly proportional to the nuclear charge and thus to the atomic number, the spin‐orbit coupling increases with the atomic number. Therefore, molecules containing heavy atoms often show a significant increase in rates of spin‐forbidden transitions, known as the heavy atom effect (HAE) [59]. Accordingly, the three 7‐X‐FAD samples show the expected behavior: The greater the molar mass of the halogen, the larger the HAE, and thus the more phosphorescence from the excited triplet state is detected.

Despite significantly altered photoreduction rates, the deprotonation rates extracted from the TA spectra barely differed, so that it can be assumed that the formation of RP2 follows a similar time constant in all samples (Table 1). While the decay kinetics of unmodified DmCry can be described with one rate constant, which corresponds to the recombination of RP2, the analyses of the three DmCry(7‐X‐FAD) samples had to be performed at two wavelengths, as two distinctly different rates were obtained. This is due to the fact that the effective photoreduction led to a significant formation of 7‐X‐FAD^·−^ in the course of the experiment, while the second rate, which reflects either the rereduction of TrpD^·^ or the recombination of RP2, remained unchanged. As the latter rate is the same for all halogenated samples and no external electron donor was added, the more efficient photoreduction could be explained by a better stabilization of the FAD^·−^ state. This in turn would shift the two competing reactions of RP2, the RP recombination and the reduction reaction of Trp_D_^·^ (Fig. 1B), towards the latter reaction. In this context, it should be noted that the increased triplet yield should in principle also be visible in the TA spectra of the DmCry(7‐X‐FAD) samples. However, a closer look at the regions above 650 nm does not show any change in absorbance compared to DmCry(FAD) [60]. The reason for this may be that the intensity of the probe lamp decreases dramatically above 650 nm and therefore minor changes in this wavelength range are difficult to detect. In addition, the triplet lifetime could be shorter than microseconds, so that in any case only a small fraction would be visible in the TA spectra.

### Origin of the altered photochemistry

Why is photoreduction so effectively enhanced by the incorporation of halogenated FAD cofactors? Analysis of the protein structure [11, 12] shows that the interactions of the 7α methyl group with its protein environment are minor and therefore derivatization at position 7α has no relevant effect on protein‐cofactor interactions.

**Fig. 8:**
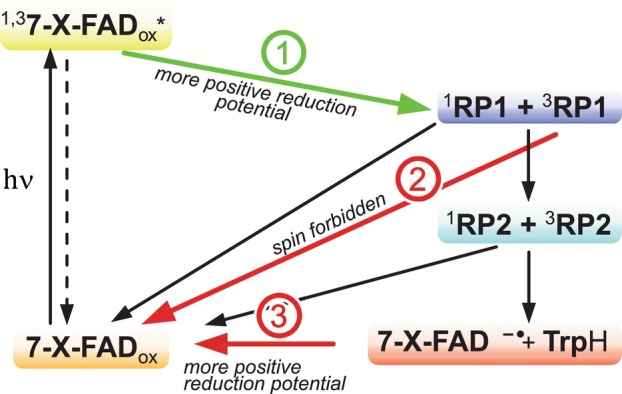
Simplified reaction scheme of changes of the reaction rates and efficiencies in DmCry(7‐X‐FAD) samples (generated using affinity designer 1.10). Red: reduced reactivity, green: enhanced reactivity.

The reason for the enhanced photoreduction (outlined in Fig. 1C) of the three halogenated DmCry(7‐X‐FAD) variants can be deduced from the spectroscopic data. Emission spectroscopy shows that a significant fraction of the excited‐state 7‐X‐FAD_ox_ cofactor is transformed to the triplet state (^3^[7‐X‐FAD*]) as the HAE significantly increases the ISC probability. Given that unmodified DmCry only forms a small fraction of triplet states, values for triplet generation yields and lifetimes are unavailable, but ISC rates of 2.7 ns^−1^ have been determined in a related photoreceptor protein [61]. This is a similar rate in which ET occurs in DmCry [37] and other Crys [62], so it can be assumed that both excited states (^1^[7‐X‐FAD*] and ^3^[7‐X‐FAD*]) are long‐lived enough to undergo ET. The intermediately formed spin‐correlated RP1 can oscillate between its singlet and triplet states driven by anisotropic hyperfine couplings [7], which is also the most accepted explanation for the magnetoreceptor properties of Crys [7]. On average, a larger fraction of RP1 may be present in its triplet configuration with halogenated flavin cofactors due to the HAE. RP1 recombination is spin‐dependent and can only take place if the spins of the two unpaired electrons are in an overall singlet state. Thus, the larger amount of ^3^RP1 directly decreases the probability of RP1 recombination (reaction 2 in Fig. 8) and thereby increases the probability of RP2 formation. RP2 in DmCry(7‐X‐FAD) can now either recombine back to the ground state, or the Trp_D_^·^ radical is reduced by neighboring, yet unknown electron donors during the lifetime of RP2. In this context, it must be noted that the absorption properties of the RPs are independent of their spin state, so they cannot be distinguished by their TA spectra. Assuming that the reduction rate constant of the Trp_D_^·^ radical is identical, a larger fraction of Trp_D_ and consequently also of FAD^·−^ is formed. The direct relation between the triplet state fraction and the increase of the photoreduction rate constant is depicted in Fig. 5A. These rates rise in correlation with the atom number of the halogen atom, with DmCry(7‐Br‐FAD) showing the most pronounced effect.

In contrast to DmCrys harboring 7‐halogenated flavin derivatives, the DmCry(7‐demethyl‐FAD) variant has a slightly lower triplet yield compared to DmCry(FAD) but shows about twice as fast photoreduction. Therefore, the triplet yield cannot be the only reason for the increased photoreduction rate. The analysis of the reduction potentials (Table 2) shows that all the modified flavins utilized have a more positive reduction potential compared to unmodified flavin, and a linear relation between the reduction potential and the increase of the photoreduction rate constant can be derived (Fig. 5B). The reduction potentials of the flavin derivatives bound in DmCry were also measured using the same methodology, but the obtained values turned out unreliable due to irreversible behavior. Nevertheless, it can be assumed that the protein‐bound flavin derivatives have similar positive differences in reduction potentials as in aqueous solution, and that the altered reduction potentials have a direct influence on the photoreduction rate: forward ET is enhanced by the more positive reduction potential, assuming that the excited modified *FAD state also has a more positive reduction potential than *FAD (reaction 1 in Fig. 8). In addition, the more positive reduction potential slows down the reoxidation of FAD^·−^ to FAD_ox_ as long as the oxidizing agent remains oxygen, resulting in a longer lifetime of the signaling state (reaction 3 in Fig. 8). The difference between DmCry(7‐demethyl‐FAD) and DmCry(7‐X‐FAD) is that in the former sample, only the altered reduction potential has an influence on the photoreduction rate, whereas in the latter samples both the increased triplet yield and the more positive reduction potential contribute to and positively influence the photoreduction rate.

The interplay of the three reaction changes outlined in Fig. 8 has the effect that both the photoreduction is significantly accelerated and the FAD^·−^ state is stabilized. Even if the biological signaling activity cannot be determined with the molecular spectroscopies used, we assume that the modified cofactors do not affect the conformational activation at the CTT, as the amino acids that interact directly with the CTT are unchanged, especially the important amino acid His378.

In conclusion, the photochemistry of DmCry could be significantly altered by the incorporation of 7‐X‐FADs, although the overall chemical modification of the isoalloxazine ring system is minor. This makes position 7α very suitable for modifying the photochemistry without altering the electronic structure of the intermediate radical pair, as was determined by the analysis of the trEPR spectra (Fig. 7). It can be assumed that further modifications, e.g., at the C8α position, will lead to further variations in the photochemistry. Since both the triplet yield and the reduction potential are linearly related to the change in photochemical kinetics, a wide range of modulation can be achieved by a clever choice of flavin modification. The use of such multi‐modified flavins is likely to alter the spin density distribution of the intermediate radical pairs, which would further change both the spin chemistry and the photochemistry. In addition to the electron acceptor, DmCry could also be modified on the electron donor side by incorporating unnatural amino acids, although the stability of such modified ET tetrads and the lower sample yield could be a challenge despite established expression systems [38].

The directed and reproducible modulation of the photoreaction in DmCry presented here can also be applied in other cryptochromes and in other light‐active flavoproteins. Because all flavin‐dependent photoreceptors are used as optogenetic tools in addition to their natural purpose [63, 64], the knowledge gained here can be used to optimize the photoresponse of an optogenetic construct for any application without the need to use variants of the protein.

## Materials and methods

### Synthesis of modified riboflavin derivatives

The RF derivatives 7‐demethyl‐RF, 8‐demethyl‐RF, 7‐F‐RF, 7‐Cl‐RF, and 7‐Br‐RF were synthesized and purified using a protocol described in Ref. [65].

### Production of DmCry samples with modified FAD cofactors

RF derivatives were incorporated into DmCry by using the riboflavin‐auxotrophic *E. coli* (*CpXribF*) strain described previously [41]. The pET28a: DmCry (C‐ and N‐terminal Histag) plasmid [17] was chemically transformed into *E. coli* (*CpXribF*) cells. Subsequent overnight protein expression at 28 °C in LB medium supplemented with 50 μg·mL^−1^ of kanamycin (Carl Roth, Karlsruhe, Germany) was carried out as described previously [41]. After harvesting (7000 ***g***, 15 min), cells were suspended in buffer A (50 mm HEPES pH 7.0, 100 mm NaCl, 10% (v/v) glycerol) and disrupted using a microfluidizer (Microfluidics, Westwood, CA, USA). Cell debris was removed by centrifugation (12 000 ***g***, 15 min) and by filtration (Rotilabo syringe filter PVDF, 0.45 μm (Carl Roth)).

### Protein purification

A Ni‐NTA column (HisTrap HP, 5 mL, GE Healthcare, Chicago, IL, USA) was equilibrated with 5 times the column volume of binding buffer (50 mm HEPES pH 7.0, 200 mm NaCl, 20 mm imidazole, 20% (v/v) glycerol). The cell extract was loaded onto the column with a flow rate of 5 mL·min^−1^. Bound protein was eluted using elution buffer (50 mm HEPES, pH 7.0, 200 mm NaCl, 500 mm imidazole, 10% (v/v) glycerol) at 65% of elution buffer. Yellow fractions were collected, concentrated (50 kDa MWCO, Amicon Ultra‐15 (Merck Millipore Darmstadt, Germany); 4000 ***g***, 4 °C, 90 min), and stored in buffer B (50 mm HEPES, pH 7.0, 100 mm NaCl, 20% (v/v) glycerol) at −70 °C. The subsequent size exclusion column (Hi Load 16/60, Superdex 200, GE Healthcare) was equilibrated with 120 mL buffer B. After sample loading, the column was developed with a flow rate of 1 mL·min^−1^, and yellow protein fractions were collected and concentrated. Some samples required additional purification. For such cases, an anionic exchange column (BabyBioTM Q, 5 mL, Bio‐Works, Victor, NY, USA) was used. The column was equilibrated using 5 times the column volume with binding buffer KCl (50 mm HEPES pH 7.0, 50 mm KCl, 10% (v/v) glycerol). The sample in binding buffer KCl was loaded onto the column, and the protein was eluted at 25% of elution buffer KCl (50 mm HEPES pH 7.0, 1 m KCl, 10% (v/v) glycerol) with a flow rate of 5 mL·min^−1^. The concentrated protein was immediately frozen in liquid nitrogen and stored at −70 °C until further use.

### HPLC analysis of denaturated DmCry variants

Concentrated protein samples were denatured using 9 m GuHCl at 60 °C for 2 min and immediately cooled on ice. The released cofactor was separated by centrifugation at 12 000 ***g*** for 1 min and filtered through a 0.45 μm syringe filter (Filtropur S 0.45, Sarstedt, Nümbrecht, Germany). The supernatant was applied to a reverse‐phase HPLC column (Vertex Plus AX column 150 × 20 mm, Eurospher II 100‐15 C18, Knauer, Berlin, Germany). Flavins were separated using a gradient of (10–35)% acetonitrile for 22 min with a flow rate of 10 mL·min^−1^. Collected fractions were immediately stored at 4 °C for further analysis (^1^H‐NMR and UV–Vis spectroscopy).

### UV–vis spectroscopy and photoreduction experiments

Absorption spectra were recorded using a UV–Vis spectrophotometer (Shimadzu UV2450, Kyoto, Japan). Samples for photoreduction experiments were prepared in buffer PR (20 mm Tris, 20 mm HEPES, 20 mm BisTris pH 7.0, 100 mm NaCl, 20% (v/v) glycerol, and 10 mm EDTA). The cuvette, buffer, and protein samples were degassed by bubbling with argon gas. The empty sealed cuvette (105.250‐QS, Hellma, Müllheim, Germany) was first degassed under argon gas for 1 h before injecting the protein sample (concentration: 50 μm). Subsequently, the filled cuvette was degassed for another hour. The cuvette was placed into a temperature‐controlled sample holder within the spectrometer, and the temperature was set to (277.0 ± 0.5) K using a flow‐through thermostat (F20‐HC, Julabo, Seelbach, Germany). The samples were illuminated using a high‐power LED (LuXEON Rebel LXML PR01 0226, Philips Lumileds Lighting Company, Schiphol, the Netherlands) emitting light at (455 ± 5) nm with a spectral irradiance of (60 ± 6) μW·cm^−2^·nm^−1^. Before measurements, a spectrum of the protein in the fully oxidized state was recorded. Samples were then illuminated with blue light at different time points until full conversion to the FAD^·−^ state was reached. To follow the progression of the photoreduction, a UV–Vis spectrum (300–700) nm was measured after each illumination cycle. All photoreduction experiments were performed under the same experimental conditions. The fractions of FADH^·^ were calculated by determining the dark state protein concentration (FAD_ox_) at 450 nm (*ε*_450_ = 11 300 m ^−1^·cm^−1^), then after completion of the reaction, the concentration of FADH^·^ was determined by the absorbance change at 580 nm (*ε*_580_ = 4800 m ^−1^·cm^−1^) [66], assuming the complete conversion of FAD^ox^ into FAD^·−^ and FADH^·^.

### Emission spectroscopy

For emission spectroscopy (LS55 Luminescence Spectrometer, Perkin‐Elmer, Waltham, MA, USA), protein samples were prepared in buffer B (50 mm HEPES pH 7.0, 100 mm NaCl, and 20% (v/v) glycerol). 400 μL of the protein sample with a concentration of 50 μm was used for measurements and placed in a fluorescence cuvette (H115F, Hellma). To follow changes in the FAD emission, spectra between 460 and 900 nm were first recorded in the dark, then samples were illuminated with blue light from a high‐power LED lamp (60 μW·cm^−2^·nm^−1^ LuXEON Rebel LXML Pr01 0226, Philips Lumileds Lighting Company) at defined time points, and subsequently, spectra were recorded. All fluorescence spectroscopy experiments were performed under identical experimental conditions. The triplet state fractions were calculated as a percentage ratio of the phosphorescence emission at 655 nm (corrected to the maxima of the phosphorescence of each flavin molecule) compared to the fluorescence emission at 520 nm (corrected to the maxima of the fluorescence of each flavin molecule).

### Electrochemistry

Cyclic voltammograms were recorded with an SP‐300 potentiostat (Bio‐Logic Science Instruments, Göttingen, Germany) to measure the half‐wave potentials of the flavin derivatives. The working electrode was a glassy carbon electrode with a diameter of 2 mm, while the reference and counter electrodes were Ag/AgCl (saturated KCl buffer) and a Pt wire, respectively. Before each measurement, the working electrode was polished with diamond paste with a particle size of 0.25 μm. Prior to measurement, the respective flavin solutions (100 mm HEPES buffer, pH 7.4) were purged with argon for at least 15 min and had a concentration of approximately 0.1 mm.

### Transient absorption spectroscopy

Transient absorption measurements were performed using a commercial laser flash spectrometer (LP920K, Edinburgh Instruments, Livingston, UK). The measurements were performed in a quartz glass cuvette (108F‐QS, Hellma) with a sample concentration of 50 μm in TA buffer (20 mm Tris, 20 mm HEPES, 20 mm BisTris pH 7.0, 100 mm NaCl, 20% (v/v) glycerol) and a volume of 600 μL at a temperature of 277 K. Samples were excited using a pulsed Nd:YAG laser (Surelite‐I, Continuum, Gilching, Germany) with a pulse frequency of 10 Hz. The pulse length was around 6 nm and a pulse energy of (3 ± 0.2) mJ at the excitation wavelength of 450 nm was used. The spectra were recorded in a wavelength range of (300–700) nm at 4‐nm steps. Some low‐stability samples were examined by measurements at larger wavelength steps to avoid protein degradation. Different time windows were chosen depending on which RP kinetics were to be analyzed (40 μs in the case of RP1, or 6 ms in the case of RP2). The primary data of the photodetector were recorded by a digital oscilloscope (TDS‐3012C, Tektronix, Berkshire, UK) and summarized with the help of the control software. In addition to the excitation spectrum, a probe‐pulse background was measured in the 10‐ms measurements and subtracted from the probe excitation measurement. As the RP kinetics are strongly influenced by the experimental conditions, e.g., the glycerol concentration or the pH value [21], all measurements were performed at standardized experimental conditions.

### Sample preparation for transient EPR spectroscopy

All samples for trEPR measurement were prepared in 100 μL of trEPR buffer (50 mm HEPES pH 7.0, 150 mm NaCl, 50% (v/v) glycerol) under aerobic conditions, and 10% K_3_[Fe(CN)_6_] equivalents of the sample concentration were added before measurement. Samples were pipetted into quartz glass EPR tubes (inner diameter of 3.1 mm and outer diameter of 3.9 mm) at a final concentration of 0.75 mm.

### Transient EPR spectroscopy

A home‐built X‐band spectrometer based on a commercial spectrometer (ESP380E, Bruker, Billerica, MA, USA) was used for transient EPR spectroscopy, which was operated in combination with a microwave bridge (ER046 MRT, Bruker) and a dielectric resonator (ER4118X‐MD5, Bruker). A laboratory‐made nitrogen gas flow cryostat (ESR935, Oxford Instruments, Abingdon, UK) was used to cool the resonator, which was kept at a constant temperature (270.0 ± 0.1) K by a temperature controller (ITC503, Oxford Instruments). A laser pulse with a wavelength of 450 nm was used for excitation, generated by an Nd:YAG laser (NT342B‐20, Ekspla, Vilnius, Lithuania) with a pulse energy of (3.0 ± 0.2) mJ, a pulse frequency of 10 Hz, and a pulse length of approximately 4 ns. Furthermore, a frequency counter (5352B, Keysight Technologies, Santa Rosa, CA, USA) was used to control the microwave frequency, having an intensity of 1.99 mW. Signal acquisition was done using a transient recorder (9354A, Teledyne LeCroy, Chestnut Ridge, NY, USA) with a bandwidth of 25 MHz. For each measurement, 200 magnetic field points were recorded in a range from 338 to 348 mT at 0.1‐mT steps, and a background that was recorded at 280 mT after every 10 measurements was subtracted. Every time trace has a length of 20 μs and contains 5000 points; 20–30 time traces were accumulated, depending on the signal intensity.

### DFT calculations

DFT calculations were performed using the orca program package (version 5.0.3) [67]. The FAD was truncated to FMN since the influence of adenosine monophosphate on the electronic properties of the isoalloxazine core is negligible. A micro‐solvated FMN model (structure 3'S_0_) was used as the input structure for FMN [68]. Atoms were modified using avogadro (version 1.0.2) to yield the respective 7‐halogenated derivatives [69]. Geometry optimization was performed with the b3lyp [70] functional using the def2‐TZVP basis set [71]. Hyperfine coupling constants and Mulliken spin populations were calculated using the B3LYP functional [70] in conjunction with the IGLO‐III basis set [72]. Since the IGLO‐III basis set does not cover the heavy atom bromine, the Partridge‐2 basis set was used for all 7‐halogen atoms [73]. In all calculations, def2/J was chosen as an auxiliary basis [74]. An atom‐pairwise dispersion correction was applied to account for dispersion forces [75] while the COSMO approach was used to account for solvation effects [76].

## Conflict of interest

The authors declare no conflict of interest.

## Author contributions

MO, AB, SW, and ES designed the research and conceived the experiments. MO prepared all samples and conducted all optical and EPR experiments. JC and AA synthesized the modified flavins. MO and ES analyzed and interpreted the spectroscopic data. EJ and VR conducted the electrochemical experiments. AS performed the DFT calculations. The figures were generated by MO, SW, and ES. The manuscript was written through the contributions of all authors. All authors have reviewed and approved the manuscript.

## Supporting information


**Fig. S1.** Modified FAD cofactors used in this study.
**Fig. S2.** UV–Vis spectra of modified FADs.
**Fig. S3.** HPLC and ^1^H‐NMR analysis of 7‐demethyl‐FAD.
**Fig. S4.** UV–Vis analysis of the photoreduction of modified DmCry samples.
**Fig. S5.** UV–Vis spectra of photoreduced DmCry(7‐X‐FAD) samples.
**Fig. S6.** Reoxidation kinetics of DmCry(7‐Br‐FAD).
**Fig. S7.** Fluorescence analysis of DmCry(FAD) compared to DmCry(7‐X‐FAD) samples.
**Fig. S8.** 2D spectra of TA measurements (6 ms time window) of DmCry(FAD) compared to DmCry(7‐X‐FAD).
**Fig. S9.** 2D spectra of TA measurements (6 ms time window) of DmCry(demethyl‐FAD) samples.
**Fig. S10.** 2D spectra of TA measurement (40 μs time window) of DmCry(FAD) compared to DmCry(7‐X‐FAD) samples.
**Fig. S11.** 2D spectra of TA measurement (40 μs time window) of DmCry(−demethyl‐FAD) samples.
**Fig. S12.** Selected 1D TA spectra of DmCry(7‐X‐FAD) samples.
**Fig. S13.** Fluorescence analysis of FAD^·−^ accumulation in DmCry.
**Fig. S14.** Normalized 2D Tr‐EPR spectra of DmCry samples.
**Table S1.** Overview of the excitation and emission properties of modified flavin derivatives.
**Table S2.** DFT‐calculated isotropic hyperfine coupling constants (in MHz) of the isoalloxazine moiety atoms for the anion radical of the 7‐halogenated flavins.

## Data Availability

The data that support the findings of this study are available within the article and the Supporting Information.
