# Supplementary material for: Linear free energy relationship between reduction potential and photoreduction rate: studies on Drosophila cryptochrome
Source: FEBS J. 2025 May 15;292(16):4254–71. doi: 10.1111/febs.70129 (PMC12366272; doi:10.1111/febs.70129)
Supplement: Supplementary file 1 — Fig. S1. Modified FAD cofactors used in this study. Fig. S2. UV–Vis spectra of modified FADs. Fig. S3. HPLC and 1H‐NMR analysis of 7‐demethyl‐FAD. Fig. S4. UV–Vis analysis of the photoreduction of modified DmCry samples. Fig. S5. UV–Vis spectra of photoreduced DmCry(7‐X‐FAD) samples. Fig. S6. Reoxidation kinetics of DmCry(7‐Br‐FAD). Fig. S7. Fluorescence analysis of DmCry(FAD) compared to DmCry(7‐X‐FAD) samples. Fig. S8. 2D spectra of TA measurements (6 ms time window) of DmCry(FAD) compared to DmCry(7‐X‐FAD). Fig. S9. 2D spectra of TA measurements (6 ms time window) of DmCry(demethyl‐FAD) samples. Fig. S10. 2D spectra of TA measurement (40 μs time window) of DmCry(FAD) compared to DmCry(7‐X‐FAD) samples. Fig. S11. 2D spectra of TA measurement (40 μs time window) of DmCry(−demethyl‐FAD) samples. Fig. S12. Selected 1D TA spectra of DmCry(7‐X‐FAD) samples. Fig. S13. Fluorescence analysis of FAD·− accumulation in DmCry. Fig. S14. Normalized 2D Tr‐EPR spectra of DmCry samples. Table S1. Overview of the excitation and emission properties of modified flavin derivatives. Table S2. DFT‐calculated isotropic hyperfine coupling constants (in MHz) of the isoalloxazine moiety atoms for the anion radical of the 7‐halogenated flavins. [file FEBS-292-4254-s001.pdf]

Linear free energy relationship between reduction potential and photoreduction rate: studies on *Drosophila* cryptochrome

Moustafa Okasha[1], Jing Chen[1], Audrey Ayekoi[1], Eike Jacob[2], Valentin Radtke[2], Anton Schmidt[1], Adelbert Bacher[3], Stefan Weber[1], Erik Schleicher[1]

1- Institute of Physical Chemistry, University of Freiburg, Germany.
2- Institute of Inorganic Chemistry, University of Freiburg, Germany.
3- TUM School of Natural Sciences, Technical University of Munich, Germany.

Supporting Information

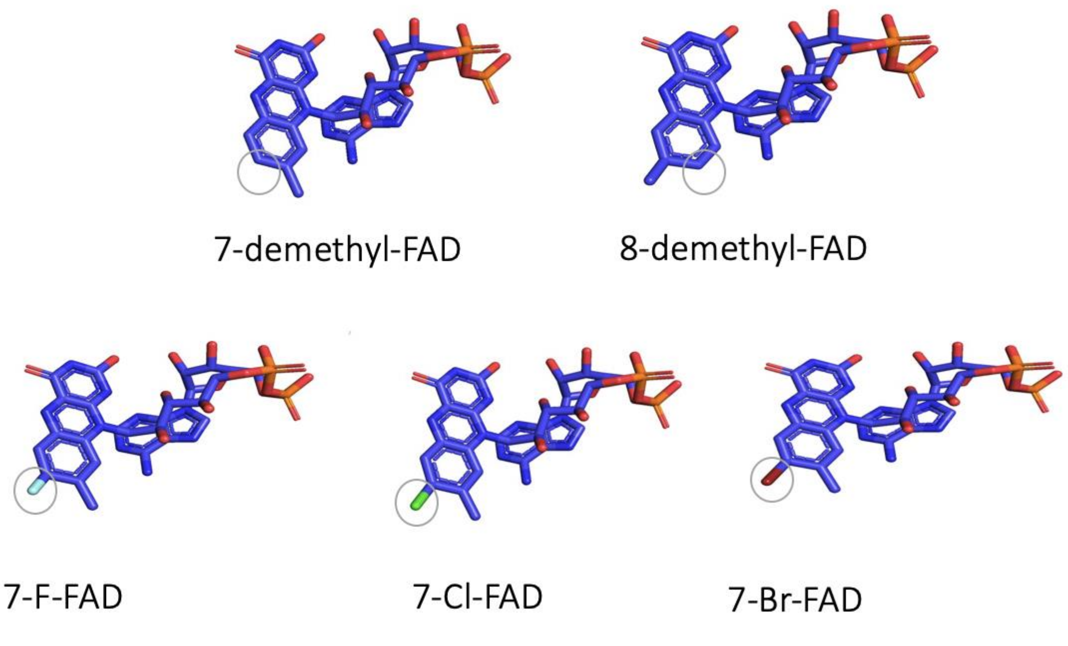

Supporting Figure 1. Modified FAD cofactors used in this study. 7-demethyl-FAD, 8-demethyl-FAD, 7-F-7-demethyl-FAD, 7-Cl-7-demethyl-FAD and 7-Br-7-demethyl-FAD.

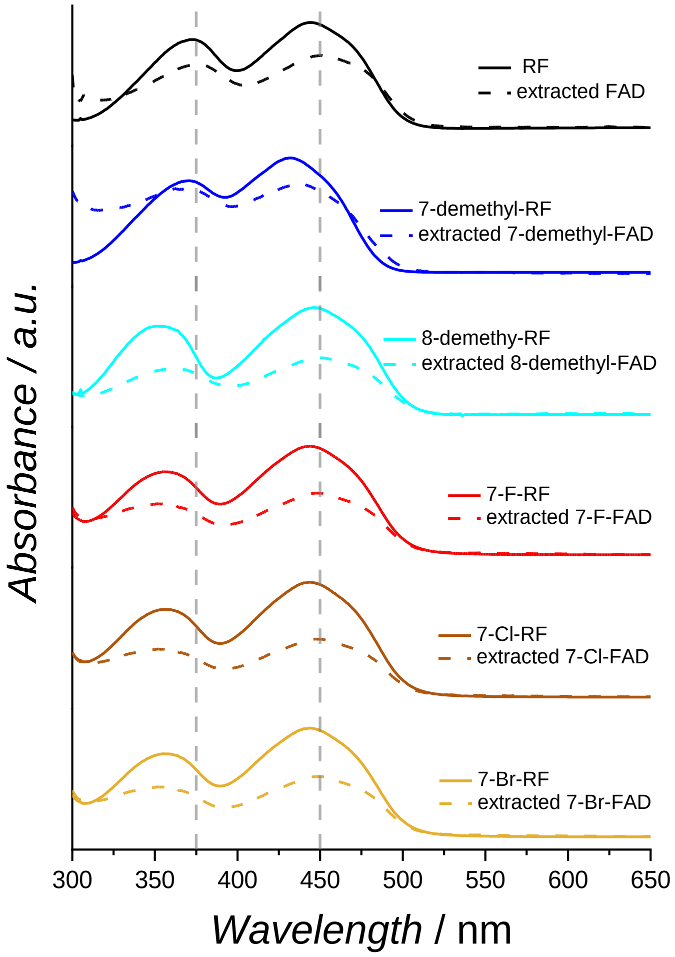

Supporting Figure 2. UV-Vis spectra of modified FADs. FADs were extracted from DmCry samples (dashed lines) and compared to the respective free RF derivatives (solid lines), and to RF and extracted FAD. The FAD cofactor was extracted with 9 M GuHCl at 42 °C in each case.

Supporting Table 1. Overview of the excitation and emission properties of modified flavin derivatives. The free flavins are in RF form, while the protein-bound flavins are in FAD form. The wavelengths of the short-wavelength ($S_0 \rightarrow S_2$ transition) maxima are indicated in brackets.

	**Free RF** λ_{ex} **/ nm**	**DmCry(FAD)** λ_{ex} **/ nm**	**Free RF** λ_{em} **/ nm**	**DmCry(FAD)** λ_{em} **/ nm**
**RF/FAD**	448 (375)	450 (376,360)	522	519
**7-demethyl-RF/FAD**	434 (370)	437 (356,374)	511	509
**8-demethyl-RF/FAD**	448 (354)	450 (344,362)	525	526
**7-F-RF/FAD**	445 (358)	449 (344,362)	521	518
**7-Cl-RF/FAD**	446 (357)	451 (362,366)	517	512
**7-Br-RF/FAD**	446 (357)	452 (362,370)	517	512

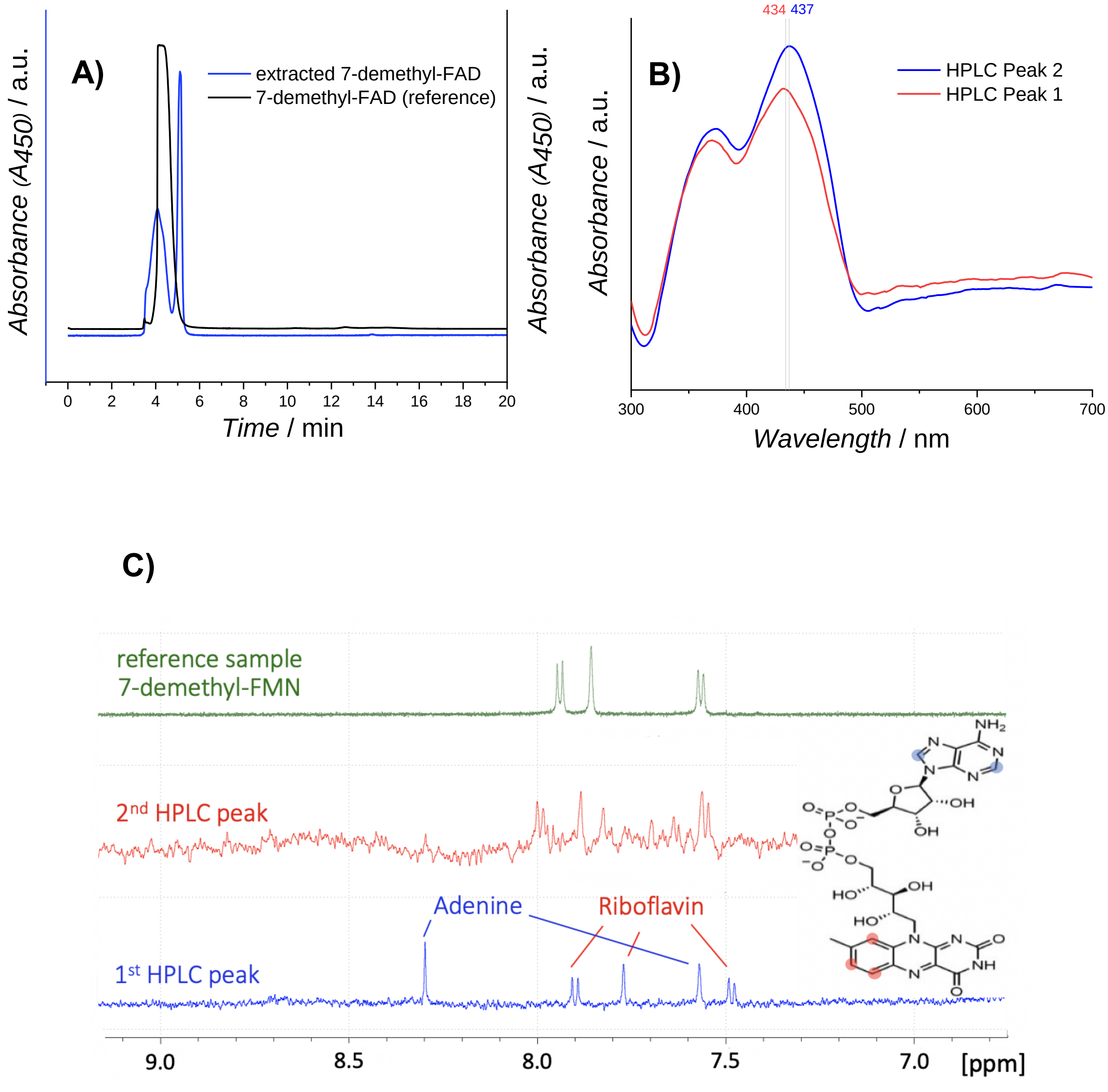

Supporting Figure 3. HPLC and ^{1}H-NMR analyses of 7-demethyl-FAD. **A)** The HPLC chromatogram of 7-demethyl-FAD extracted from DmCry(7-demethyl-FAD) shows two fractions with slightly different retention times (4–5 min). **B)** UV-Vis spectra of the two HPLC fractions. **C)** ^{1}H-MNR aromatic region spectra of HPLC fractions compared to a reference spectrum of 7-demethyl-FMN identify the two fractions as 7-demethyl-FAD and 7-demethyl-FMN.

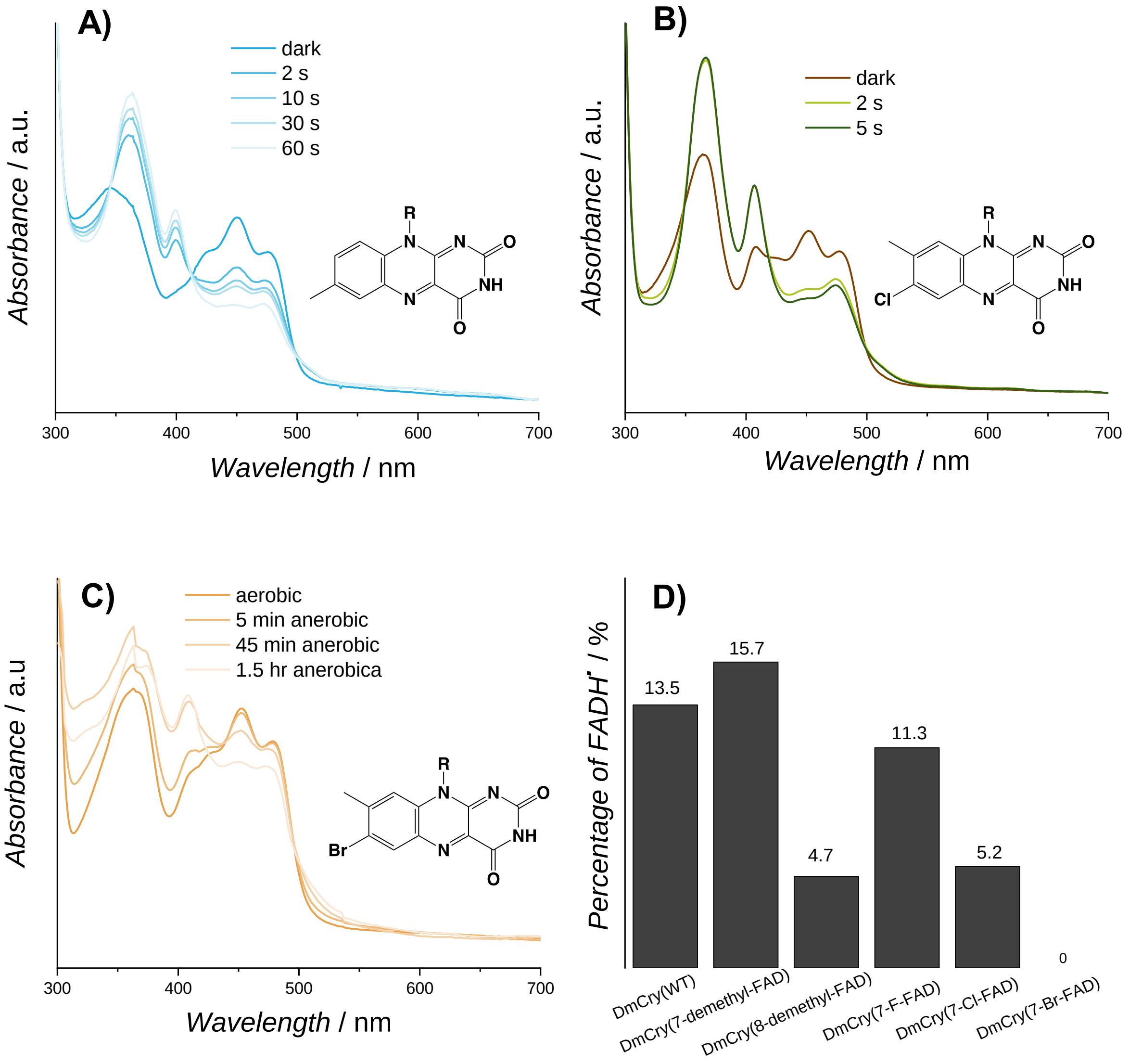

Supporting Figure 4. UV-Vis analysis of the photoreduction of modified DmCry samples. **A)** DmCry(8-demethyl-FAD); **B)** DmCry(7-Cl-FAD); **C)** DmCry(7-Br-FAD) reduction under anaerobic conditions in the dark. Note that the absorption change at 372 nm is an artefact of the spectrometer. **D)** Fraction of neutral FADH$^{\bullet}$ in photoreduced samples determined via the calculation of the (620/450) nm absorbance ratio.

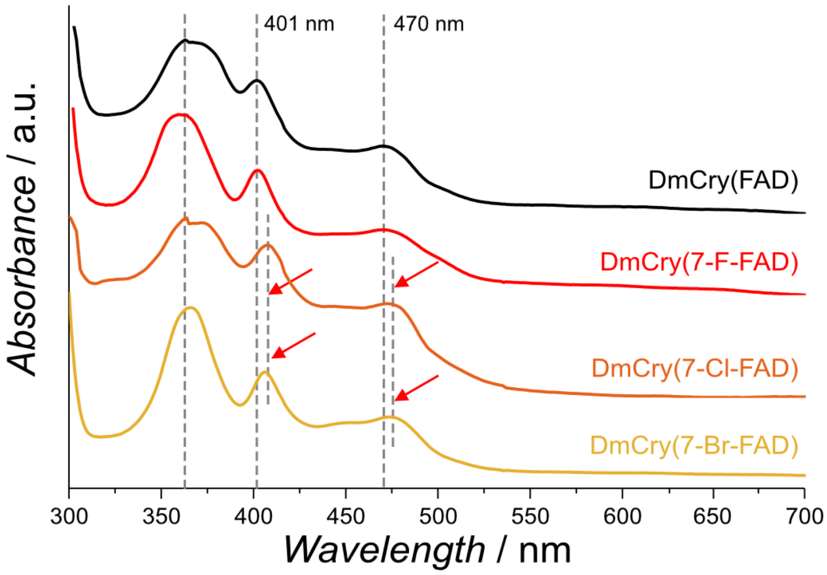

Supporting Figure 5. UV-Vis spectra of photoreduced DmCry(7-X-FAD) samples. Selected maxima of $FAD^{\bullet -}$ and differences between samples are highlighted.

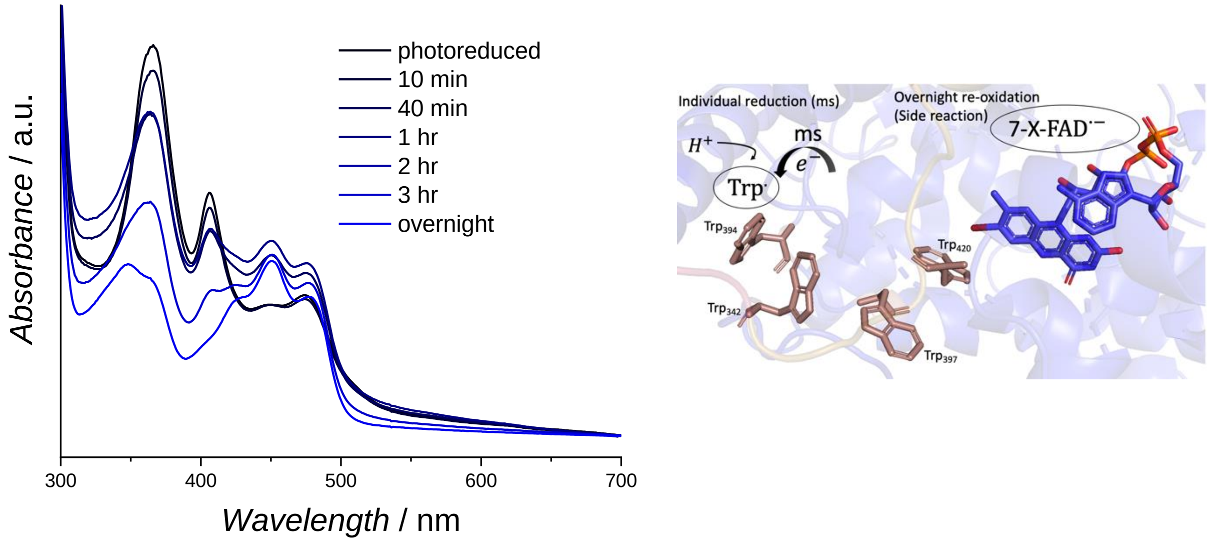

Supporting Figure 6. Reoxidation kinetics of DmCry(7-Br-FAD). The series of UV-Vis spectra show the slow aerobic reoxidation from the signaling state ($FAD^{\bullet -}$) to the dark state (FAD_{ox}) in DmCry(7-Br-FAD), the reoxidation kinetics of the other two DmCry(7-X-FAD) samples is similar. The sample was anaerobically photoreduced without an external electron donor and then incubated under aerobic conditions. The spectra were recorded at the indicated time points.

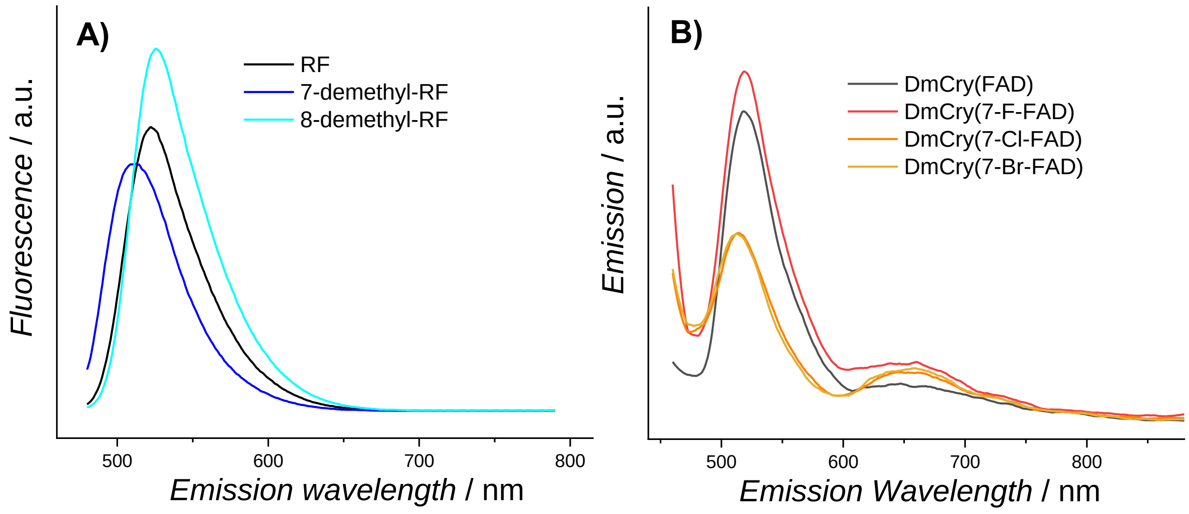

Supporting Figure 7. Fluorescence analysis of DmCry(FAD) compared to DmCry(7-X-FAD) samples**. A)** Fluorescence spectra of 7-demethyl-RF and 8-demethyl-RF compared to RF. **B)** Emission spectra of DmCry(7-X-FAD) samples compared to DmCry(FAD). All protein samples had the same concentration.

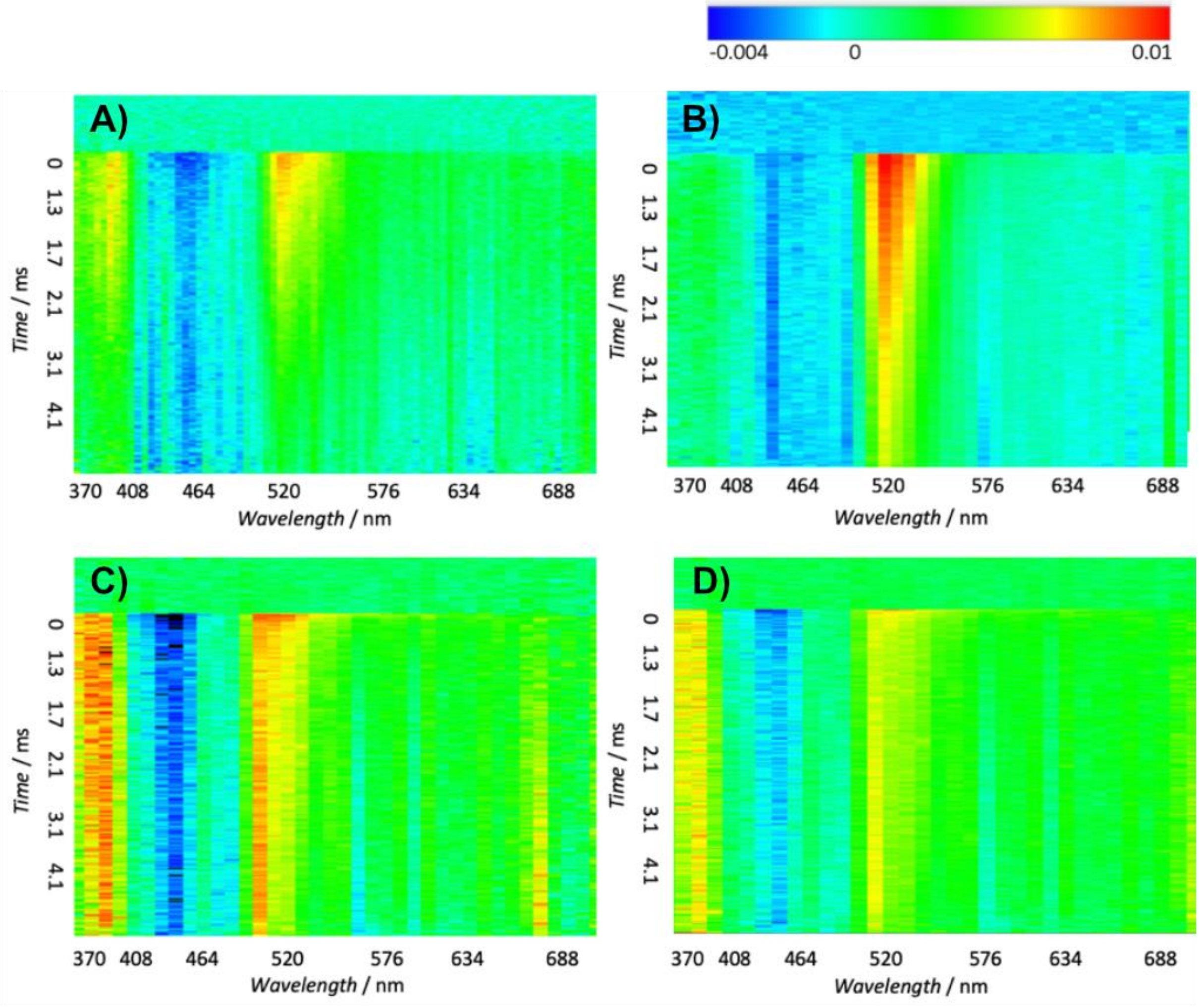

Supporting Figure 8. 2D spectra of TA measurements (6 ms time window) of DmCry(FAD) compared to DmCry(7-X-FAD). **A)** DmCry (FAD), **B)** DmCry(7-F-FAD), **C)** DmCry(7-Cl-FAD), and **D)** DmCry(7-Br-FAD). The color code ranges from –0.004 (blue) to 0.01 (red).

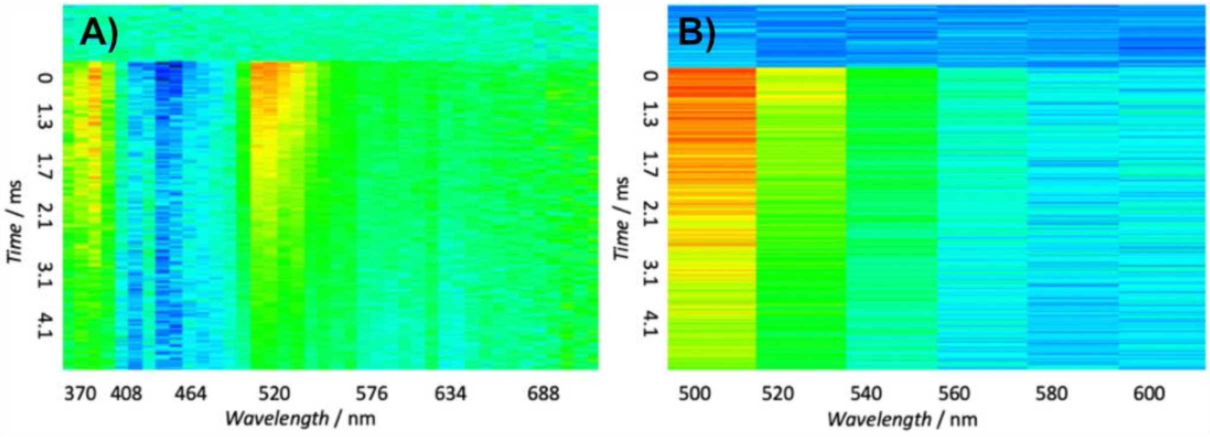

Supporting Figure 9. 2D spectra of TA measurements (6 ms time window) of DmCry(demethyl-FAD) samples. **A)** DmCry(7-demethyl-FAD), and **B)** DmCry(8-demethyl-FAD). The TA spectrum of DmCry(8-demethyl-FAD) was recorded only at the indicated wavelength window (500–600) nm with a 20 nm interval to account for the reduced sample stability and hence, to minimize sample degradation. The color code ranges from –0.004 (blue) to 0.01 (red).

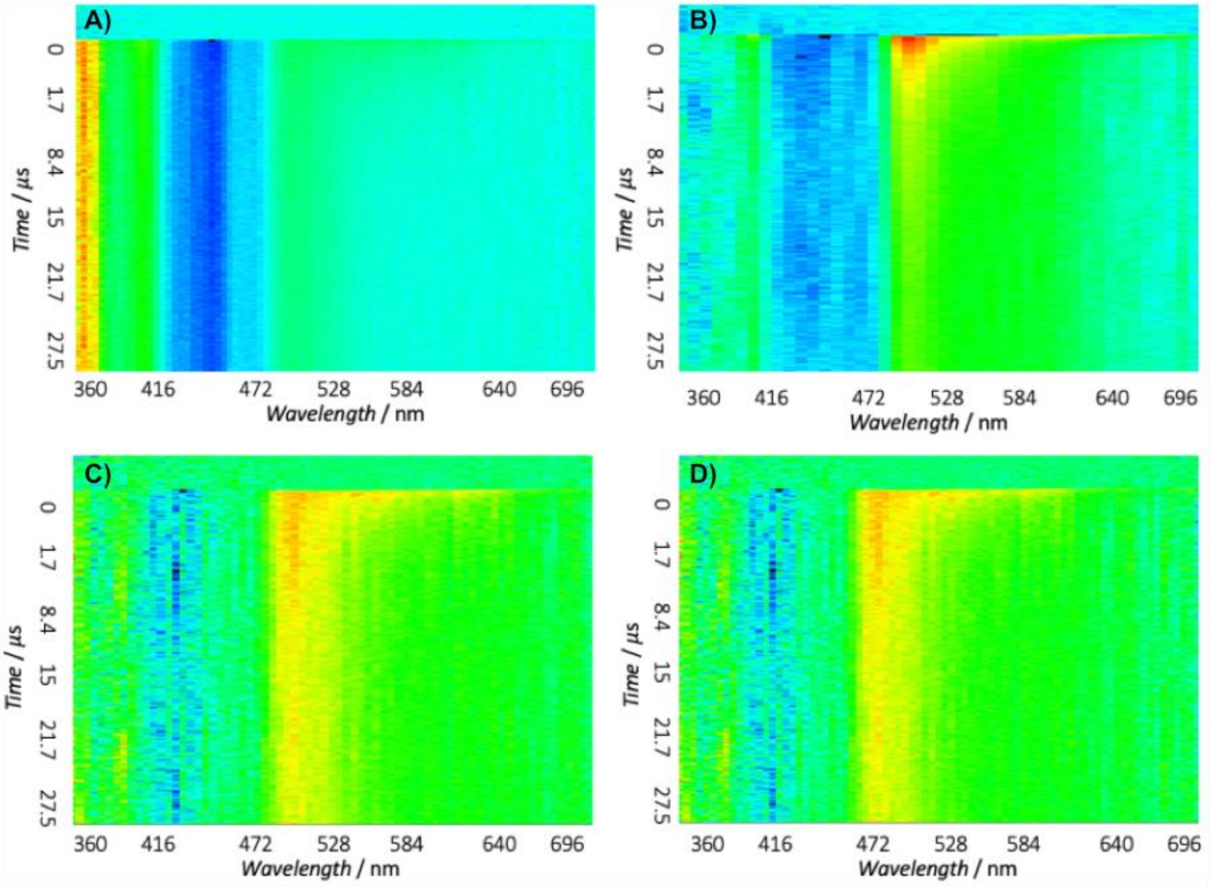

Supporting Figure 10. 2D spectra of TA measurement (40 µs time window) of DmCry(FAD) compared to DmCry(7-X-FAD) samples. **A)** DmCry(FAD), **B)** DmCry(7-F-FAD), **C)** DmCry(7-Cl-FAD), and **D)** DmCry(7-Br-FAD). The color code ranges from –0.004 (blue) to 0.01 (red).

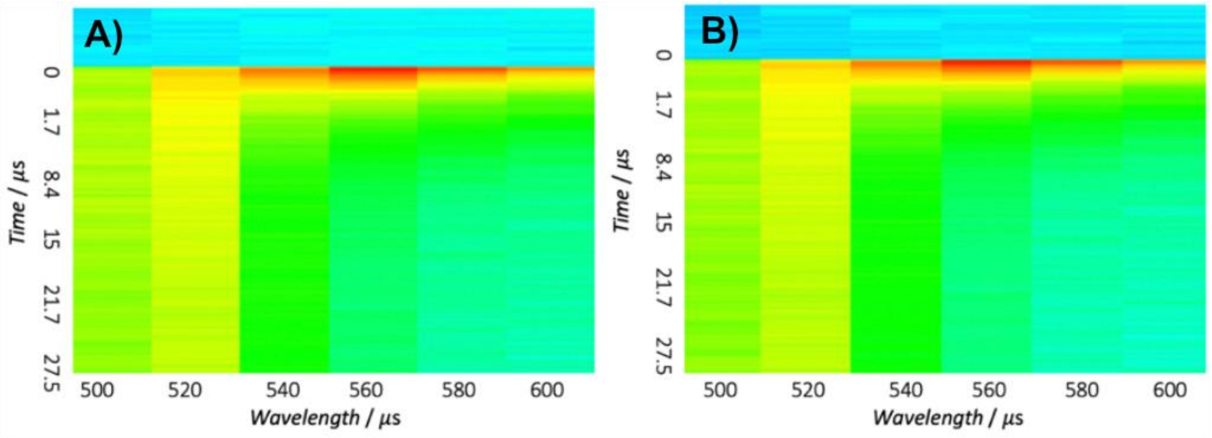

<u>Supporting Figure 11:</u> 2D spectra of TA measurement (40 µs time window) of DmCry(-demethyl-FAD) samples. **A)** DmCry(7-demethyl-FAD), and **B)** DmCry(8-demethyl-FAD). TA spectra were recorded at the indicated wavelength window (500–600 nm) with a 20 nm interval to account for the reduced sample stability and hence, to minimize sample degradation. The color code ranges from –0.004 (blue) to 0.01 (red).

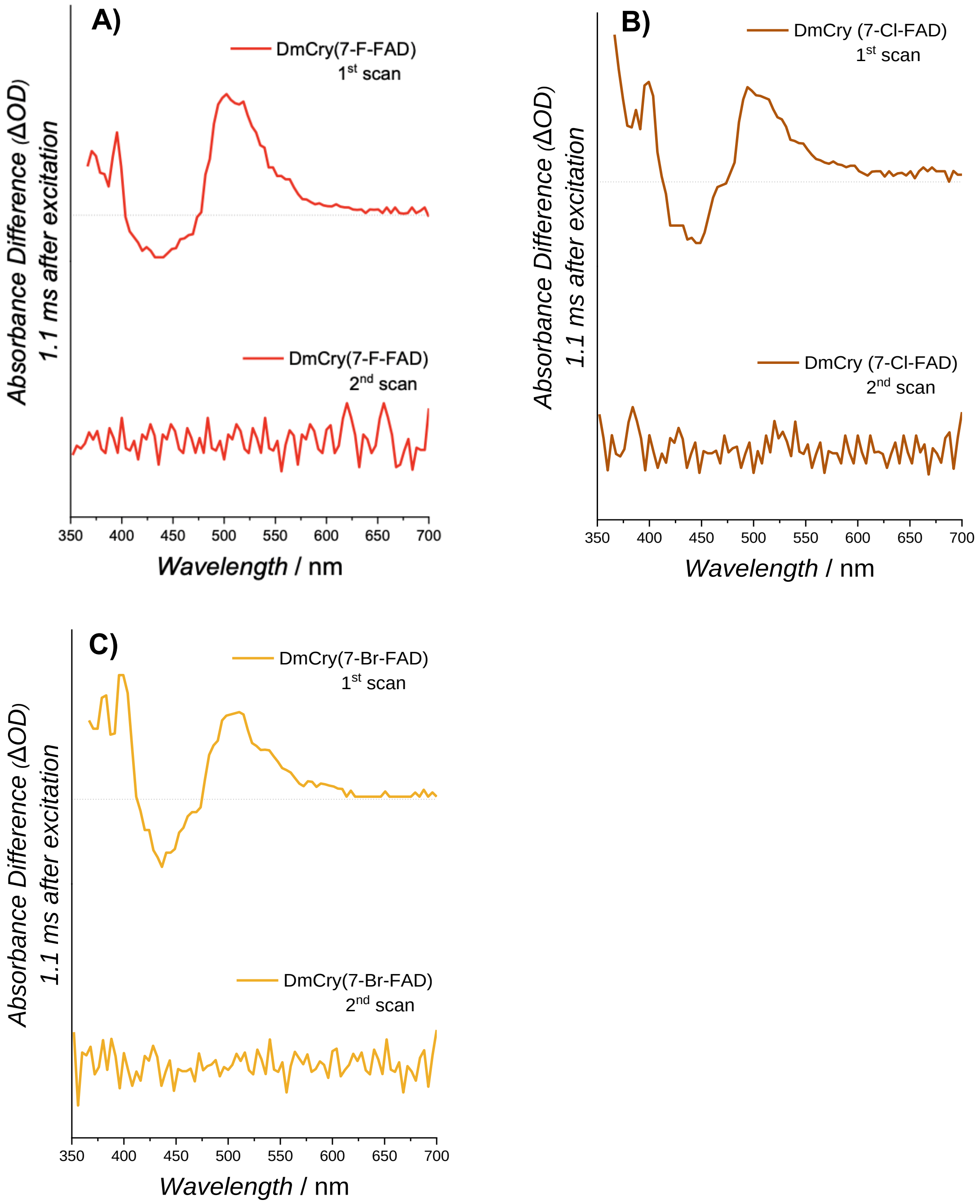

Supporting Figure 12. Selected 1D TA spectra of DmCry(7-X-FAD) samples. **A)** DmCry(7-F-FAD), **B)** DmCry(7-Cl-FAD), and **C)** DmCry(7-Br-FAD). Two subsequent measurements are shown; clearly, no absorbance differences can be detected in the second scan due to the accumulation of $FAD^{\bullet-}$.

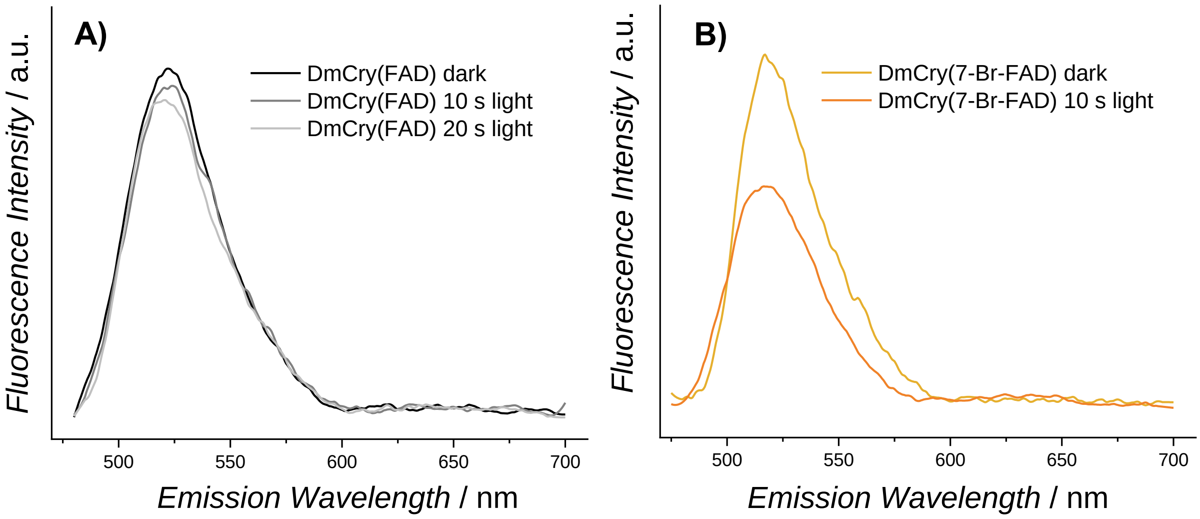

Supporting Figure 13. Fluorescence analysis of $FAD^{\bullet-}$ accumulation in DmCry. **A)** Fluorescence spectra of DmCry(FAD) in the dark and after 10 s and 20 s of blue-light illumination. **B)** Fluorescence spectra of DmCry(7-Br-FAD) in the dark and after 10 s of blue-light illumination reveal a significant reduction of the fluorescence intensity due to the accumulation of $FAD^{\bullet-}$.

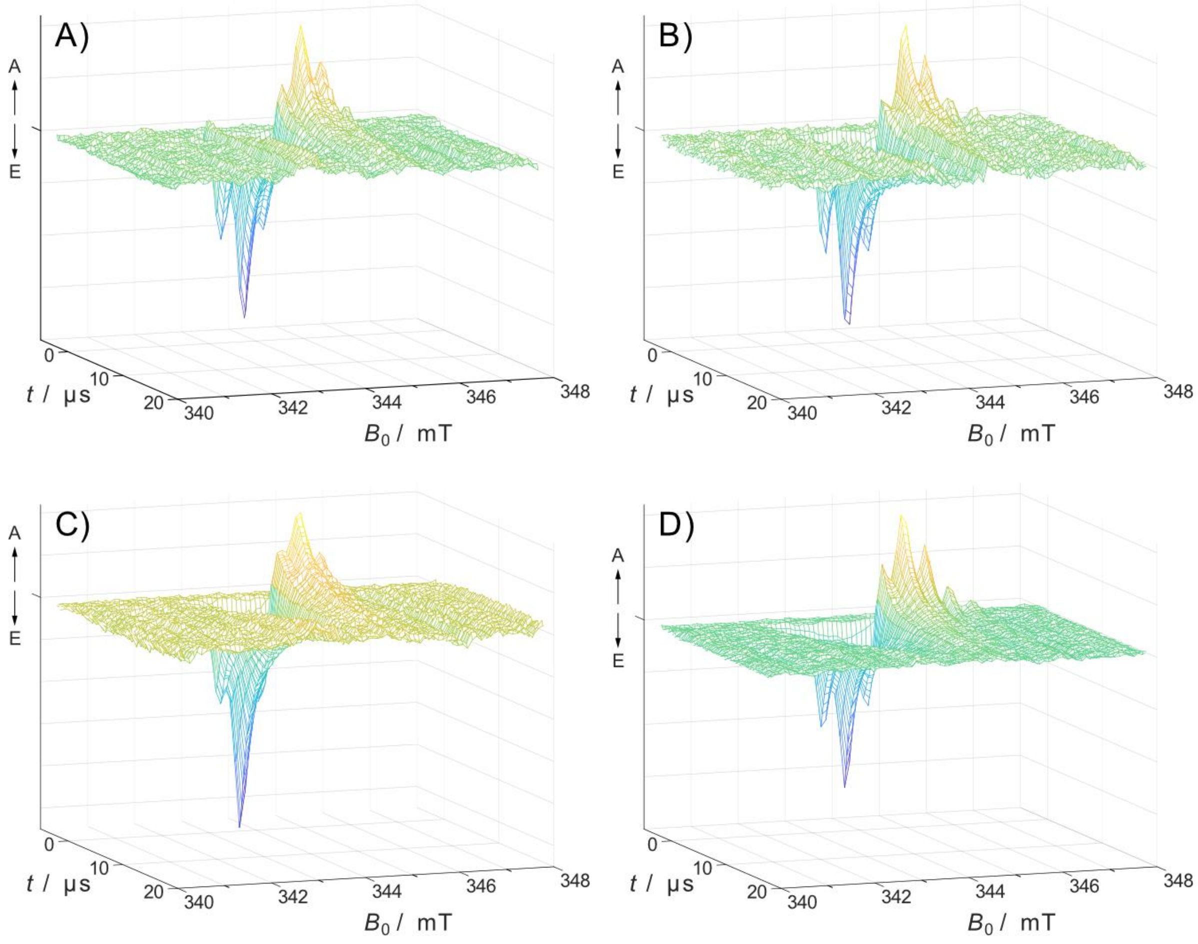

Supporting Figure 14. Normalized 2D Tr-EPR spectra of DmCry samples. **A)** DmCry(FAD), **B)** DmCry(7-F-FAD), **C)** DmCry(7-Cl-FAD), and **D)** DmCry(7-Br-FAD). All spectra were shifted to a microwave frequency of 9.65308 GHz.

Supporting Table 2. DFT-calculated isotropic hyperfine coupling constants (in MHz) of the isoalloxazine moiety atoms for the anion radical of the 7-halogenated flavins. Isotropic hyperfine coupling constants of the three hydrogen atoms in the 8α-position were averaged, X7 denotes the respective halogen in the 7-halogenated FMN derivatives.

	Isotropic hyperfine couplings / MHz		
**Nucleus**	**7-F-FMN**	**7-Cl-FMN**	**7-Br-FMN**
**X7**	–6.57	–0.57	–1.12
**H3**	–0.80	–0.73	–0.72
**H6**	–7.76	–8.41	–8.63
**H8α**	11.83	11.08	10.87
**H9**	2.14	2.03	2.01
**C2**	0.88	0.94	0.96
**C4**	–1.33	–1.26	–1.25
**C4a**	–8.62	–9.06	–9.15
**C5a**	–22.18	–22.26	–22.31
**C6**	9.33	10.10	10.32
**C7**	–12.01	–12.09	–12.12
**C8**	13.69	13.24	13.06
**C8α**	–5.64	–5.35	–5.26
**C9**	–10.41	–9.70	–9.50
**C9a**	6.64	5.82	5.60
**C10a**	–6.76	–6.26	–6.12
**N1**	–0.87	–0.85	–0.87
**N3**	–0.94	–1.00	–1.01
**N5**	16.54	16.78	16.83
**N10**	7.07	6.90	6.86
**O2**	–2.76	–2.90	–2.93
**O4**	–4.61	–4.61	–4.60
